# Global impact of the COVID-19 lockdown on biodiversity data collection

**DOI:** 10.1038/s41598-025-93275-z

**Published:** 2025-03-13

**Authors:** Stephanie Roilo, Jan O. Engler, Anna F. Cord

**Affiliations:** 1 Chair of Computational Landscape Ecology, TUD Dresden University of Technology, Helmholtzstr. 10, 10169 Dresden, Germany; 2 Agro-Ecological Modeling Group, Institute of Crop Science and Resource Conservation, University of Bonn, Niebuhrstr. 1a, 53113 Bonn, Germany; 3 AviCon - Forschung & Planung, 90765 Fürth, Germany

**Keywords:** Birdwatching, Citizen science, Coronavirus, eBird, GBIF, Pandemic, Ecological modelling, Ecology

## Abstract

The COVID-19 pandemic triggered different governmental responses across borders, with cascading effects on people’s movements and on biodiversity data collection. We quantified changes in the number of species occurrence records collected during the first global lockdown (March 15th to May 1st 2020) relative to pre-pandemic levels using data from the Global Biodiversity Information Facility (GBIF). We modelled how such changes relate to the stringency of governmental policy responses, changes in human mobility, and countries’ population size and economic class across 129 countries. We further focused on data from the community science project eBird, which constitutes the largest dataset in GBIF, to investigate changes in participation and activity patterns of individual observers (eBirders) during the lockdown. We found that the decreases in GBIF records correlated with declines in numbers of visitors to parks and outdoor areas, and were significantly larger in developing countries compared to developed ones. While the activity ranges of eBirders shrunk across all countries analysed, the number of eBirders in developing and least developed countries declined more than in developed countries, as the lockdown disrupted the influx of international visitors. Our results suggest that community-based, local monitoring programmes are essential to reduce biases in global biodiversity monitoring.

## Introduction

The COVID-19 pandemic dramatically disrupted human activities and mobility across the globe^1,2^. In an attempt to contain the spread of the virus, governments imposed travel restrictions and lockdowns that triggered an “anthropause”^2^. During the peak of the pandemic, approximately 57% of the world’s population faced travel restrictions^3^. Amidst its human and economic impacts, the pandemic hence offered a unique chance to “quantify the effects of human activity on wildlife”^2^.

Yet, because biodiversity data is primarily gathered through direct human observation^4^, travel restrictions limited data collection and consequently the quantification of biodiversity responses to the reduction in human activity. Field surveys were interrupted^4,5^, and community science projects were affected by fluctuations in participation rates^6–11^. For example, surges in reporting activity were documented in urban areas in the United States, Italy, Spain and the United Kingdom during the lockdown^6,7,10^. Meanwhile, Sweet et al.^8^ observed a general increase in community monitoring participation in Bavaria, Germany, but found no significant changes in observation rates across varying levels of urbanisation. For southeast Australia, Stenhouse et al.^12^ found that observation counts from a national community science platform were not affected by the lockdown, whereas Kishimoto & Kobori^9^ reported a drop in participation rates to the 4-day-long bioblitz event City Nature Challenge which took place in April 2020 in Tokyo. More recently, a global analysis of birdwatching data^11^ showed that reporting rates decreased more in middle and low-income regions compared to high-income ones, where participation levels recovered quickly after the lockdown.

Existing literature clearly shows that the effects of the COVID-19 lockdown on biodiversity data collection were highly variable across countries. Yet, a comprehensive evaluation of how country-specific regulations affected people’s movements and data collection activities is lacking. At the global level, the main drivers explaining increases or decreases in collected data remain unclear. The only global assessment so far focused solely on data from the eBird project^11^, while more and more biodiversity monitoring projects are collating open-source data on a variety of taxa^13^.

Here, we used data from the Global Biodiversity Information Facility (GBIF), the world’s largest biodiversity data network^14^, to calculate changes in the amount of species occurrence records collected during the lockdown relative to pre-pandemic levels. We focused on the first global COVID-19 lockdown period, i.e. March 15th to May 1st 2020. During this period, over half of the world’s population was under mobility restrictions^3^. We related the change in number of biodiversity records to the stringency of governmental responses to the pandemic and to changes in human mobility. Using data from 129 countries and dependent territories, we tested whether stricter lockdown regimes, and their consequent effects on human mobility, correlate with reductions in volumes of biodiversity data. We additionally considered country-specific factors likely to influence data collection, such as population size and economic development^15^. Pre-existing temporal trends in GBIF data in each country were accounted for by modelling data volumes over the years 2010–2023 and comparing the predicted to the actual number of records collected during the 2020 lockdown.

Moreover, to understand how movement restrictions affected participation rates and activity patterns of individual observers, we restricted our analysis to the most popular community science project and largest dataset in GBIF, eBird^13,16^. The eBird dataset includes unique identifiers of each observer, hereafter eBirder, allowing us to quantify changes in participation to eBird, and to anonymously track eBirders’ movements and activities through the georeferenced bird records they contributed. We expected to find both increases and decreases in participation to the platform depending on the country’s lockdown policy. More specifically, we hypothesised that the number of eBirders would decrease in countries where particularly strict lockdown measures prevented or discouraged individuals to engage in biodiversity-recording activities outdoors, or where the inflow of eBirders from other countries (e.g. tourists, researchers, etc.) was disrupted by the lockdown measures. We also expected eBirders to have much smaller activity ranges (i.e. to survey smaller areas) during the lockdown, especially in countries with the strictest movement restrictions.

## Results

### Impact of COVID-19 restrictions on biodiversity data collection

**Fig. 1:**
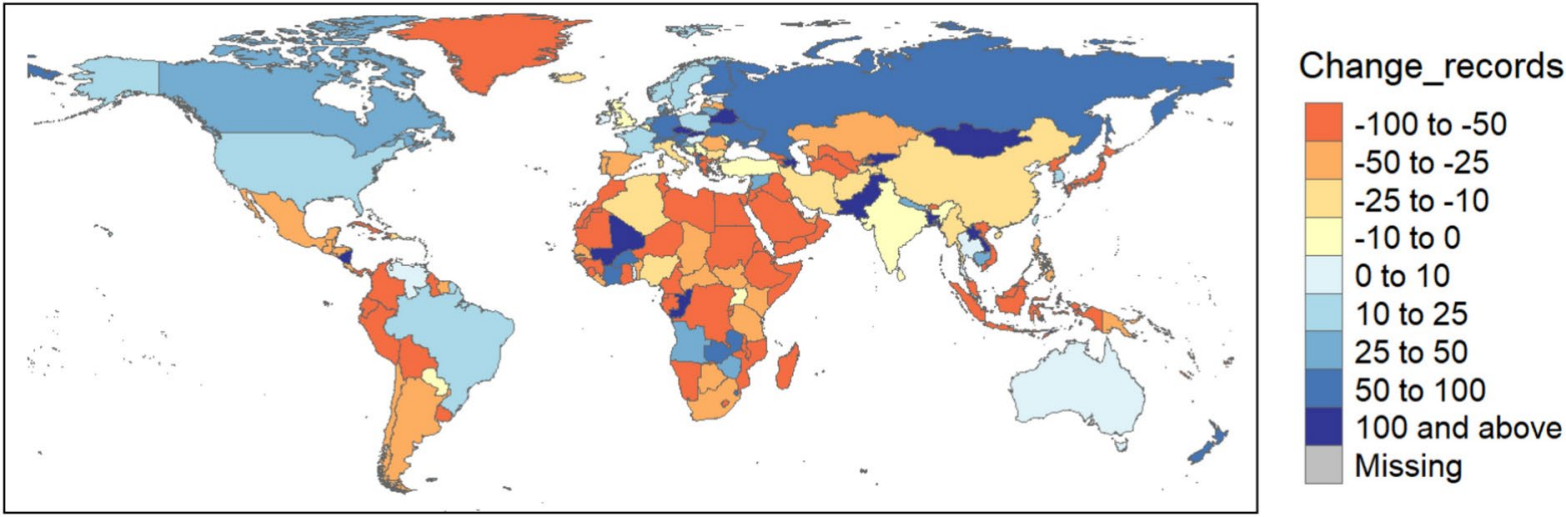
Percent change in mean daily number of GBIF records collected between March 15th and May 1st 2020 relative to the same period in 2019. *Change_records* could not be calculated for Bouvet Island, Heard Island and McDonald Islands, and Nauru, as there were no GBIF records dated between March 15th and May 1st 2019. The dataset can be explored interactively in the Anthropause_app (https://sroilo.shinyapps.io/Anthropause_app/), which also allows to calculate *Change_records* for different time periods.

The change in GBIF records (*Change_records*) collected during the lockdown period in 2020 as compared to the same period in 2019 was highly variable across countries (Fig. 1). *Change_records* was negative for most countries in Latin and Central America, Africa and southern Asia (with some exceptions, e.g. Bangladesh, Brazil, Mali, Nepal, Pakistan, Thailand, Togo, Zambia, Zimbabwe), and in southern Europe (Fig. 1, Table S1). Contrarily, in many northern and central European countries, as well as in the United States, Canada, Australia and New Zealand, *Change_records* was positive, indicating that more GBIF records were collected during the 2020 lockdown compared to the same period in the previous year.

For the majority of countries, the number of GBIF records collected during the lockdown was significantly (p < 0.05) lower than what would be expected based on the trends in data volumes over the years 2010 to 2023 (Fig. S1a-b, Table S2). Notably, in certain countries, the number of records collected during the lockdown significantly exceeded the predicted data amount; this was the case in many northern European countries (e.g. Denmark, Finland, Germany, Norway, Sweden) but also in countries with historically lower data volumes in GBIF (e.g. Bangladesh, Mongolia, Pakistan and Zambia; Fig. S1c, Table S2).

**Fig. 2:**
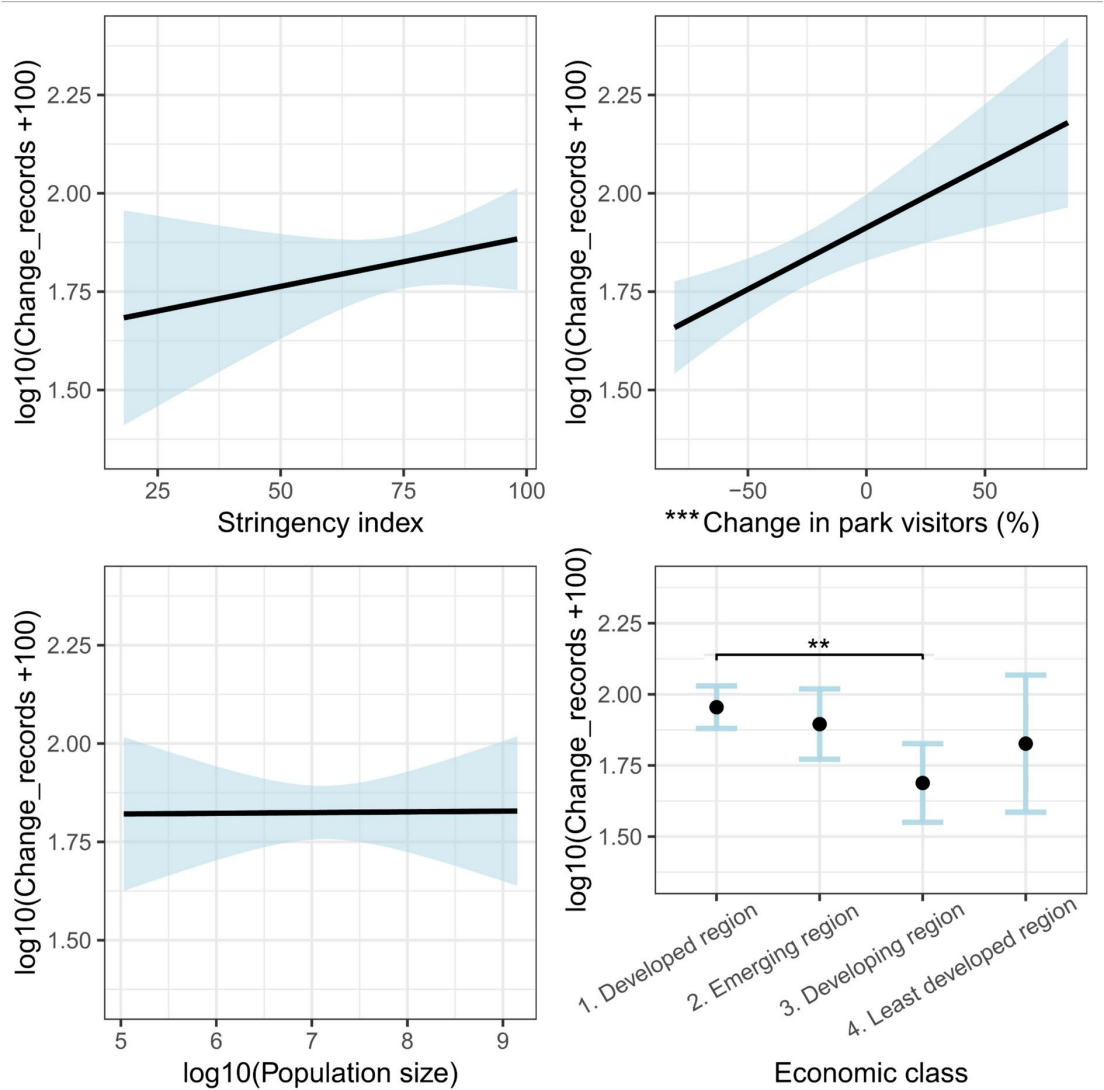
Conditional plots of the predictors of the linear regression model, extended using the generalised least squares method. Relationships were graphed for each predictor with all other continuous covariates held at their means, or by weighting levels of the categorical covariate in proportion to sample size. The light-blue shading shows the confidence interval at the 0.95 level. Asterisks indicate significance of the estimated regression coefficient of the predictor at the 0.01 (**) and 0.001 (***) levels.

The Generalised Least Squares regression identified two significant predictors of *Change_records* across countries (Fig. 2): the change in the number of visitors to parks and outdoor areas (*Change_park_visitors*), which had a positive and significant regression coefficient (p = 0.0008); and the countries’ economic class (*Economic_class*), for which the difference between developed and developing countries was significant (p = 0.0012). *Change_records* was highest in developed countries compared to all other economic classes, though the difference was not significant for countries with emerging economies (p = 0.4331) and for least developed ones (p = 0.3155). The regression coefficients for the stringency index (*Stringency_index*) and the countries’ population size (*log10_Population_size*) were positive, but neither of the two was significant (p = 0.2925 and p = 0.9671, respectively). The addition of an interaction term between *Stringency_index* and *Economic_class* led to a slight but non-significant improvement of model fit (log-likelihood of the base model = -41.11; log-likelihood of the interaction model = -37.49, log-likelihood ratio test’s p = 0.064), and the analysis of variance found the interaction term to be non-significant (p = 0.0759). The conditional plots of the interaction model showed some evidence of a diverging effect of *Stringency_index* across economic classes, as it correlated negatively with *Change_records* in developing countries but positively in all others. However, the effects of all other predictors did not change significantly from the base model (Fig. S2).

### Impact of COVID-19 restrictions on eBirders’ numbers and activity

**Fig. 3:**
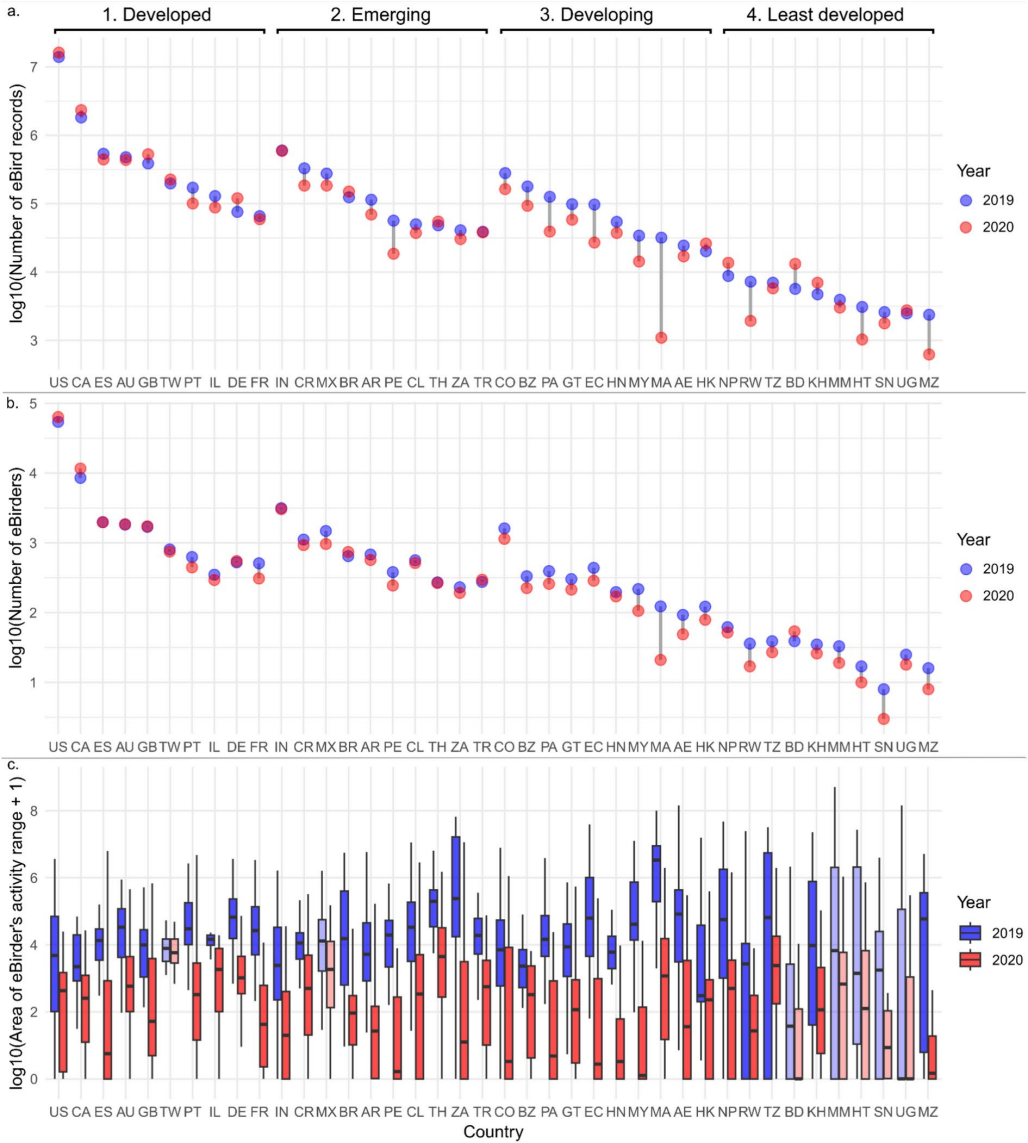
Number of eBird records (**a**) and of eBirders (**b**) between March 15th and May 1st in 2019 (blue dots) and in 2020 (i.e. during lockdown; red dots), shown by country; and area (km^2^) of the activity ranges of the 30 most active (i.e. who recorded species on the most days between March 15th and May 1st 2019) eBirders per country in the two time periods (**c**). All numbers are log10-transformed. The top ten countries or dependent territories per economic class (1. Developed, 2. Emerging, 3. Developing, and 4. Least developed; see top of the figure) with most eBird records during that time period were selected for the analysis. Country codes, countries’ full names and the number of mapped activity ranges for each country and time period are available in Table S3. Lighter shading of the boxplots indicates that the area of the activity ranges of eBirders between the two time periods is not significantly different (Kolmogorov–Smirnov test’s p-value > 0.05).

The number of eBird records collected during the lockdown decreased in most countries (28 out of 40 countries and dependent territories that were analysed; Fig. 3a), and especially in the developing ones, with the only exception of Hong Kong (HK). In turn, eBird records increased in many developed countries and dependent territories, such as the United States (US), Canada (CA), the United Kingdom (GB), Taiwan (TW) and Germany (DE) during the lockdown relative to pre-pandemic levels. Similarly, the number of eBirders increased during the lockdown in certain developed and emerging countries, but decreased across all developing and least developed countries, except for Bangladesh (BD; Fig. 3b). The activity ranges of eBirders shrunk in nearly all analysed countries (Fig. 3c), indicating that records collected from the same observer were more clustered during the pandemic. Four least developed countries, namely Haiti (HT), Senegal (SN), Uganda (UG), and Mozambique (MZ), had fewer than 30 active eBirders between March 15th and May 1st 2019, thus fewer activity ranges could be mapped, likely affecting the statistical power of the cross-year comparison in these countries (see Table S3 for the number of activity ranges mapped per country and the p-value of the Kolmogorov–Smirnov test). Indeed, the area of the activity ranges was significantly (p < 0.05) larger in 2019 than in 2020 in all but seven countries, namely Taiwan (TW), Mexico (MX), Bangladesh (BD), Myanmar (MM), Haiti (HT), Senegal (SN), and Uganda (UG; Fig. 3c).

**Fig. 4:**
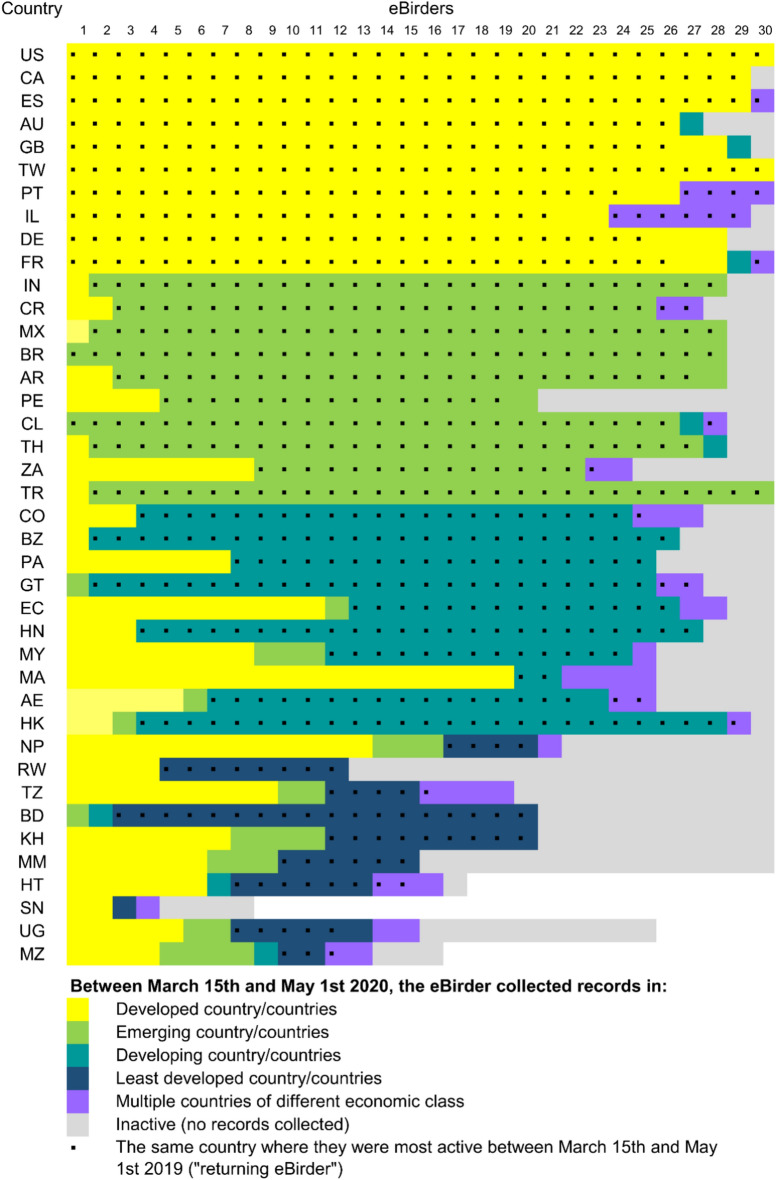
Economic class of the countries in which records were collected by the 30 most active eBirders (i.e. those who recorded species on the most days between March 15th and May 1st 2019; numbered from 1 to 30 at the top of the figure) per country or dependent territory during the lockdown in 2020. The black square symbol marks eBirders that, during the lockdown, collected records (also) in the same country where they had been selected as most active between March 15th and May 1st 2019 (i.e. the country listed on the left-hand side of the figure). Country codes and full names are listed in Table S3.

Mapping the records of individual eBirders in geographical space revealed interesting transboundary “migrations” between pre-lockdown and lockdown periods. In all ten developed countries analysed, 83% or more of the most active eBirders in the focal country between March 15th and May 1st 2019 continued to record species in the same country during the 2020 lockdown (Fig. 4). We observed a similar trend in countries with emerging economies, with the exceptions of Peru (PE) and South Africa (ZA), which had only 50% of ‘returning’ eBirders. Developing and especially least developed countries lost a large proportion of eBirders who either became inactive (i.e. stopped recording data) or spent the lockdown in other countries, typically developed ones (Fig. 4).

## Discussion

The COVID-19 pandemic had a substantial impact on data collection efforts. We recorded large cross-country variations in the amount of species observations collected globally during the lockdown compared to pre-pandemic levels. Despite the lockdown, data volumes increased in many developed countries (Fig. 1, Table S2). These gains in collected records were positively correlated with increments in park visitation during the lockdown (Fig. 2). Lockdown regulations that allowed exercising outdoors enabled an increase in outdoor recreational activities among the population. This was the case for many central and northern European countries such as Germany, Sweden and Finland, where outdoor recreational activities increased during the pandemic^17–19^, leading to positive changes in data volumes (Fig. 1^,^ Tables S2). Park visitation increased also in the United States and Canada^20,21^, while New Zealanders increased walking activities within their neighbourhood when all other outdoor activities were prohibited^22^. These changes correlated with positive changes in biodiversity data collection (Fig. 1), suggesting that when people were allowed to spend time outside, they resumed biodiversity monitoring or even joined community science projects for the first time^23,24^. Indeed, the number of eBirders increased during the lockdown relative to the prior year in countries like Australia, Canada, Germany, the United Kingdom and the United States (Fig. 3b), although this may be due to pre-existing positive trends in participation to the platform^25^, rather than a lockdown effect.

On the other hand, we uncovered reductions in data volumes in southern Europe and in most of Africa and Latin America (Fig. 1). In southern Europe, strict stay-at-home requirements in countries like Italy and Spain, where infection rates were high^6^, may be responsible for the observed declines in biodiversity data compared to other European countries (Fig. 1). The same mechanism may explain at least part of the data losses in many African countries and in Central and South America. Indeed, home confinement orders extended for weeks or even months in Ghana, Nigeria, South Africa, Sudan, Uganda and Zimbabwe^26^. Total lockdown measures, during which movements were allowed only for essential services, were adopted also in Argentina, Bahamas, Bolivia, Colombia, Ecuador, El Salvador, Haiti, Panama, Paraguay, Peru, Trinidad and Tobago, and Venezuela^27,28^. The few countries that adopted more relaxed measures (e.g. Zambia, Brazil and Nicaragua^26,28,29^ stand out as exceptions in Africa and Latin America, as they are among the limited number of countries with a positive change in species observation records (Fig. 1). In Asia, regulations varied widely from rigid home quarantine in the Hubei province in China^30^ and in the Philippines^31^ to much softer lockdown policies in Taiwan^32^ and no lockdown at all in Cambodia in 2020^33^, contributing to respectively negative and positive changes in data volumes (Fig. 1). Despite these observations, the regression models did not show any negative effect of lockdown stringency on *Change_records* (Fig. 2), except for developing countries when an interaction term between stringency and economic class was included in the model structure (Fig. S2). One possible explanation for this is that the stringency index is a composite index, reflecting not only restrictions on people’s movement, but also other governmental policies (e.g. school and workplace closures, public information campaigns)^1^ which do not directly translate to reduced mobility or access to nature. The latter is, in turn, effectively captured by the predictor *Change_park_visitors*, which had a highly significant effect in the regression model (Fig. 2). Moreover, lockdown regimes often varied greatly within the same country, as different subnational administrative units were subject to different restrictions. Increasing the spatial resolution of the analysis may thus provide a different picture of the impact of stringency levels on data collection.

The variations in lockdown stringency and the consequent changes in outdoor visitation were not the only factors explaining the observed patterns in biodiversity data collection as the pandemic unfolded. In certain countries such as Benin, Botswana, Cameroon, Fiji, Rwanda and Yemen, large declines in species observation records contrasted with only moderate decreases in outdoor activity (Table S1). We found that reductions in collected records between lockdown and pre-pandemic times were significantly larger in developing countries compared to developed ones (Fig. 2). We argue that the decrease in data volumes in developing and developed countries is due to a decline in the number of eBirders, which is reflected in a reduction in eBird records (Fig. 3a,b). As eBird records make up for the vast majority of GBIF records in many developing and least developed countries (Table S4), fluctuations in eBirders’ numbers in those countries are likely responsible for the observed GBIF data losses (Fig. 1). At the other end of the spectrum, in countries like Denmark, France, the Netherlands, Norway and Sweden, eBird records constitute only a small fraction of the total GBIF data volume (< 5%; Table S4), while other national community science platforms are popular there. For example, the Swedish Artportalen, DOF/BirdLife Denmark and the Norwegian Species Observation Service are the second, fifth, and sixth largest occurrence datasets in GBIF, respectively^34^. Without a dedicated analysis, it is impossible to evaluate whether the changes in participation to eBird that we observed are representative of trends in other (national or international) community science projects during the lockdown. To date, similar analyses only encompassed few other platforms besides eBird, namely iNaturalist^6,7,27^, eButterfly^7^, the US-based Nature’s Notebook^7^, the German Igel in Bayern^8^ and the Australian echidnaCSI^12^, and reported contrasting changes in participation and reporting activity depending on the country, the platform, and (sub)national geographic variations in lockdown restrictions.

Biodiversity records collected by individual observers were geographically more clustered compared to pre-pandemic times. Indeed, the activity range of eBirders shrunk across most analysed countries (Fig. 3c). Interestingly, this was the case also in countries with relatively relaxed lockdown regimes (e.g. Germany, Brazil, but Taiwan stands out as an exception), and in countries where participation in eBird increased (e.g. Canada, United Kingdom, United States). This shows that, irrespective of the countries’ specific lockdown regulations, eBirders’ recording behaviour was constricted globally by the pandemic.

Many of the most active eBirders in developing and least developed countries between March 15th and May 1st 2019 contributed records in different countries during the 2020 lockdown (Fig. 4). If we assume that most people spent the lockdown in their country of residence, our results suggest that the reduced data collection in the Global South may be caused by the disrupted inflow of international eBirders. This hypothesis is supported by a recent global analysis of eBird data^11^ showing that eBirders reporting from multiple countries decreased substantially during the pandemic compared to previous years. While we only analysed 30 eBirders per country, it has been shown that the vast majority of eBird data derives from few highly active participants^35^. Our results suggest that international (i.e. from abroad) tourists, researchers, and bird enthusiasts make a disproportionately larger data contribution to the GBIF platform in developing countries than in developed ones. eBird in particular is a popular community science platform in developed countries like the United States, Canada and Australia^25^, but less so among residents of developing and least developed countries, where participation is often hindered by socio-economic and technological barriers, such as lack of IT resources^36^. For example, participation in community science projects requires (at minimum) a smartphone with access to the internet^15^^,^ resources that are not evenly distributed across the globe^36,37^. On the other hand, neither GBIF nor their contributory projects offer an accurate cross-country representation of biodiversity monitoring effort and community involvement in it. In fact, a wealth of (sub)nationally-sourced datasets may be stored in local repositories rather than in international platforms like GBIF, especially if the latter requires additional efforts towards data standardization. Moreover, in certain countries the use of externally-led digital platforms for storing community-based monitoring data raises issues of data governance, potentially threatening local data ownership and Indigenous knowledge sovereignty^38,39^. Targeted promotion and support of local and national biodiversity monitoring initiatives, ideally engaging indigenous and local communities not only in data collection but also in the design, development and maintenance of data repositories, is indispensable to collect biodiversity data globally and comprehensively in the long term^40,41^.

Another notable trend in our data is the large proportion of eBirders in developing and least developed countries who stopped collecting data during the lockdown (Fig. 4). We can only speculate about the causes of such a trend, but our results suggest that data contributors were impacted differently by the pandemic depending on their profile. While passionate birders may have been forced to renounce their international birding activities to record only locally during lockdown^24^, scientists had to cancel field trips^42^, and many local bird guides went inactive due to lack of customers^43^. Our analysis of eBirders’ movements constitutes an example of how exploring the characteristics and motivations of individual observers holds great potential for an improved understanding of the impacts of future developments (pandemics, international conflicts, changes in international travel due to increased environmental awareness) on biodiversity data collection globally. In other words, to better understand biases in biodiversity data, we ultimately need to better understand the demographics of data contributors^44,45^.

## Conclusions

We showed large cross-country variations in the amount of biodiversity records collected during the COVID-19 lockdown. Overall, biodiversity data collection was significantly reduced during the pandemic in developing countries. Our analysis of eBirders’ movements suggests a credible explanation: travel restrictions stopped incoming visitors from developed countries, leading to a drop in data contributors. This implies that comprehensive and sustained data collection across borders can only be achieved through increased support to community-based, local and national biodiversity monitoring initiatives. Our insights show that understanding the distribution and dynamics of data contributors could be instrumental in helping to maintain and sustain global biodiversity data collection in the least biased way.

## Methods

### Data preparation

#### Biodiversity data

We downloaded the GBIF occurrence snapshot dated January 1st, 2025^46^, from which we extracted the number of georeferenced species records tagged as ‘human observation’ collected in different countries. We aggregated records by their country code, which distinguishes the country, territory or area in which the occurrence was recorded^47^. The list of country codes included 251 administrative units, i.e. the 249 officially recognized country codes as per the ISO 3166–1 list^48^, plus “XK” for Kosovo and “ZZ” for high seas. Records collected in high seas were excluded from further analysis as they fell outside national jurisdictions (i.e. in international waters). To quantify changes in the amount of records collected during the lockdown as compared to the previous year, we calculated the percent change in number of records (*Change_records*), expressed as

$${Change\_records} (\%) = \frac{No. of record{s}_{t1} - No. of record{s}_{t0}}{No. of record{s}_{t0}} \times 100$$

Where t1 corresponds to the time period from March 15th to May 1st 2020, i.e. the first global lockdown during which the largest proportion of people were under mobility restrictions^3^, and t0 is the period between the same dates in 2019. It is well-known that data contributions to GBIF have been increasing, often exponentially, in most countries over time^49^. To test whether the number of records collected during the 2020 lockdown differed significantly from the expected trend, we inspected country-specific counts of GBIF records between 2010 and 2023. We fitted smoothing splines to the number of records collected between March 15th and May 1st of each year, using the ss() function of the npreg package^50^ (Fig. S1). This allowed us to obtain the predicted number of records along with the lower and upper confidence interval limits for 2020, which we then compared to the actual number of records collected during the lockdown (Table S2). We repeated the analysis using three different values for the spline’s smoothing parameter, namely 0.4, 0.5, and 0.6. A lower value of the smoothing parameter allows higher flexibility of the spline. Data preparation and analyses were conducted in R version 4.4.1^51^.

#### Human mobility data

We quantified changes in human mobility during the pandemic using data from the Google COVID-19 Community Mobility Reports^52^. These data inform on patterns of human mobility at the national level. From these reports, we extracted data on the change in average time (hours) spent in places of residence (*Change_time_at_home*) and the change in the number of visitors to parks and outdoor spaces (including, e.g., public gardens, national forests, campgrounds, *Change_park_visitors*). Both variables measure percent changes compared to a five-week baseline period established before the start of the pandemic, namely between January 3rd and February 6th 2020.

#### Stringency index

We measured national governmental responses to the pandemic using the stringency index developed by Hale et al. (2021)^1^. This is a composite measure based on nine indicators, namely school closure, workplace closure, public transport closure, public events’ cancellation, restriction on gatherings, stay-at-home requirements, restrictions on internal movements, international travels’ restrictions, and public information campaigns. The index ranges from 0 (no restrictions) to 100 (strictest restrictions). The stringency index was retrieved from the COVID-19 dataset of Our World In Data^53^.

#### Population size and economic class

Population size and economic development are known to influence the amount of records contributed to GBIF^15^. Following this premise, we retrieved national data on population sizes (*Population_size*) as well as their designation in terms of economic development (*Economic_class*). We distinguished the latter into four classes: (1) Developed, (2) Emerging, (3) Developing, (4) Least developed. Population estimates were downloaded from Our World in Data^53^. The distinction of economic development classes was obtained from the R package rnaturalearth^54^.

### Global analysis of the lockdown impact on biodiversity data collection

We used linear regression models to investigate the drivers of the observed change in records. *Change_time_at_home* was highly correlated with *Change_park_visitors* (Spearman’s rho = -0.74), so we excluded it from the model. A logarithmic transformation of base 10 was applied to *Change_records* (after adding an offset of 100 to avoid negative values) and to *Population_size* to reduce the right-skewness of their distribution*.* The model formula was thus:

$$\begin{gathered} \log _{{10}} \left( {Change\_records{\mkern 1mu} + {\mkern 1mu} 100} \right){\mkern 1mu} \sim {\mkern 1mu} Stringency\_index{\mkern 1mu} + {\mkern 1mu} Change\_park\_visitors{\mkern 1mu} + \hfill \\ {\mkern 1mu} \log _{{10}} \left( {Population\_size} \right){\mkern 1mu} + {\mkern 1mu} Economic\_class \hfill \\ \end{gathered}$$

Of the 250 administrative units for which data was collated, 121 were excluded due to missing data on human mobility or stringency index. We fitted the model with the remaining 129 countries (Fig. 1, Table S1), and inspected the model residuals using the function simulateResiduals() of the DHARMa package^55^. The diagnostic plots revealed significant heteroscedasticity (Levene test for homogeneity of variance p-value = 0.002). We thus extended the linear model using Generalised Least Squares to allow different variances per category of *Economic_class* using the nlme package^56^. To test whether *Stringency_index* had a differential effect across levels of *Economic_class*, we refitted the model by adding an interaction term between the two variables, and we compared the two models by performing an analysis of variance, using the maximum likelihood method, via the anova() function of the stats package^51^. The significance of predictors in the interaction model was further assessed via an analysis of variance. To display the effect of each predictor, we produced conditional plots using the effects package^57,58^.

### Analysis of the lockdown impact on Birders’ numbers and activity

We further focused on eBird records only, as they have a unique observer identifier, allowing us to study individual data contributors. To assess how the participation in eBird changed during the lockdown, we extracted the number of eBird records collected between March 15th and May 1st in 2019 and in 2020 per country from GBIF. From those, we derived the number of active eBirders (unique observer IDs) per country in the two time periods.

To map changes in the activity range of individual eBirders, we selected ten countries per economic class which had the highest number of records collected between March 15th and May 1st 2019 (Table S4), ensuring sufficient data availability. For these countries, we identified the 30 most active eBirders in 2019, i.e., those who recorded species on the most days. In some countries, fewer than 30 eBirders were active during the selected time period, so that all of them were included in the analysis (Table S3). For each eBirder, we calculated their activity range by determining the area (in km^2^) of the minimum convex polygon that encompassed all their records from March 15th to May 1st in 2019 and in 2020, respectively. We used the Kolmogorov–Smirnov test to determine if there were significant (p-value < 0.05) differences in the area of the activity ranges between the two time periods. Finally, we compiled the list of countries where eBirders gathered records during the lockdown to investigate their cross-country movements.

## Supplementary Information

Supplementary Information.

## Data Availability

The GBIF datadet utilized in this study is available at https://doi.org/10.15468/dl.uzt932. The R code developed for the analysis is archived in Zenodo: https://doi.org/10.5281/zenodo.14772861. The collated data on the stringency index, change in visitors to parks and change in time spent at home, as well as the calculated change in records (see “Biodiversity data” in the Methods section), can be explored through an interactive webtool, the Anthropause app (https://sroilo.shinyapps.io/Anthropause_app). The app allows users to compare the variables across countries for different time periods (“Global” tab in the app), and to explore temporal trends of each variable for single countries (“Single country” tab).

## References

[1] Hale, T. et al. A global panel database of pandemic policies (Oxford COVID-19 Government Response Tracker). *Nat. Hum. Behav.* **5**, 529–538 (2021). 33686204 10.1038/s41562-021-01079-8

[2] Rutz, C. et al. COVID-19 lockdown allows researchers to quantify the effects of human activity on wildlife. *Nat. Ecol. Evol.* **4**, 1156–1159 (2020). 32572222 10.1038/s41559-020-1237-z

[3] Bates, A. E. et al. Global COVID-19 lockdown highlights humans as both threats and custodians of the environment. *Biol. Conserv.* **263**, 109175 (2021). 34035536 10.1016/j.biocon.2021.109175 PMC8135229

[4] Sugai, L. S. M. Pandemics and the Need for Automated Systems for Biodiversity Monitoring. *J. Wildl. Manage* **84**, 1424–1426 (2020). 32904967 10.1002/jwmg.21946 PMC7461419

[5] Scerri, E. M. L. et al. Field-based sciences must transform in response to COVID-19. *Nat. Ecol. Evol.* **4**, 1571–1574 (2020). 32929241 10.1038/s41559-020-01317-8

[6] Basile, M., Russo, L. F., Russo, V. G., Senese, A. & Bernardo, N. Birds seen and not seen during the COVID-19 pandemic: The impact of lockdown measures on citizen science bird observations. *Biol. Conserv.* **256**, 109079 (2021). 34580546 10.1016/j.biocon.2021.109079 PMC8457629

[7] Crimmins, T. M., Posthumus, E., Schaffer, S. & Prudic, K. L. COVID-19 impacts on participation in large scale biodiversity-themed community science projects in the United States. *Biol. Conserv.* **256**, 109017 (2021). 36531527 10.1016/j.biocon.2021.109017 PMC9746923

[8] Sweet, F. S. T., Rödl, T. & Weisser, W. W. COVID-19 lockdown measures impacted citizen science hedgehog observation numbers in Bavaria. *Germany Ecol. Evolut.* **12**, e8989 (2022). 10.1002/ece3.8989 PMC9204849 35784062

[9] Kishimoto, K. & Kobori, H. COVID-19 pandemic drives changes in participation in citizen science project “City Nature Challenge” in Tokyo. *Biol. Conserv.* **255**, 109001 (2021). 34565806 10.1016/j.biocon.2021.109001 PMC8455166

[10] Hochachka, W. M., Alonso, H., Gutiérrez-Expósito, C., Miller, E. & Johnston, A. Regional variation in the impacts of the COVID-19 pandemic on the quantity and quality of data collected by the project eBird. *Biol. Conserv.* **254**, 108974 (2021). 34629475 10.1016/j.biocon.2021.108974 PMC8486489

[11] Qiao, H. et al. Global birdwatching data reveal uneven consequences of the COVID-19 pandemic. *Biol. Conserv.* **288**, 110351 (2023).

[12] Stenhouse, A. et al. COVID restrictions impact wildlife monitoring in Australia. *Biol. Conserv.* **267**, 109470 (2022). 35136243 10.1016/j.biocon.2022.109470 PMC8814614

[13] Chandler, M. et al. Contribution of citizen science towards international biodiversity monitoring. *Biol. Conserv.* **213**, 280–294 (2017).

[14] Heberling, J. M., Miller, J. T., Noesgaard, D., Weingart, S. B. & Schigel, D. Data integration enables global biodiversity synthesis. *Proc. Natl. Acad. Sci. U.S.A.* **118**, e2018093118 (2021). 33526679 10.1073/pnas.2018093118 PMC8017944

[15] Zizka, A. et al. Bio-Dem, a tool to explore the relationship between biodiversity data availability and socio-political conditions in time and space. *J. Biogeogr.* **48**, 2715–2726 (2021).

[16] Auer, T. *et al.* EOD – eBird Observation Dataset. 10.15468/aomfnb (2024).

[17] Anke, J., Francke, A., Schaefer, L.-M. & Petzoldt, T. Impact of SARS-CoV-2 on the mobility behaviour in Germany. *Eur. Transp. Res. Rev.* **13**, 10 (2021). 38624595 10.1186/s12544-021-00469-3 PMC7835317

[18] Fagerholm, N., Eilola, S. & Arki, V. Outdoor recreation and nature’s contribution to well-being in a pandemic situation - Case Turku Finland. *Urban Forestry Urban Greening* **64**, 127257 (2021). 34493936 10.1016/j.ufug.2021.127257 PMC8414054

[19] Hansen, A. S., Beery, T., Fredman, P. & Wolf-Watz, D. Outdoor recreation in Sweden during and after the COVID-19 pandemic – management and policy implications. *J. Environ. Plan. Manag.* **66**, 1472–1493 (2023).

[20] Geng, D. C., Innes, J. L. & Wang, G. Survive, revive, and thrive: The impact of COVID-19 on global park visitation. *Sci. Total Environ.* **946**, 174077 (2024). 38908585 10.1016/j.scitotenv.2024.174077

[21] Ferguson, M. D. et al. The Outdoor Renaissance: Assessing the Impact of the COVID-19 Pandemic upon Outdoor Recreation Visitation, Behaviors, and Decision-Making in New England’s National Forests. *Soc. Nat. Resour.* **35**, 1063–1082 (2022).

[22] Espiner, N., Degarege, G., Stewart, E. J. & Espiner, S. From backyards to the backcountry: Exploring outdoor recreation coping strategies and experiences during the 2020 COVID-19 pandemic in New Zealand. *J. Outdoor Recr. Tourism* **41**, 100497 (2023). 10.1016/j.jort.2022.100497 PMC8882411 37521270

[23] Jones, K. Bird watching during coronavirus: Melbourne Facebook group takes flight. https://www.smh.com.au/lifestyle/life-and-relationships/bird-watching-takes-flight-during-the-pandemic-20200605-p54zyv.html (2020).

[24] Devokaitis, M. Lots of People Are Discovering the Joy of Birding From Home During Lockdown. *All About Birds* https://www.allaboutbirds.org/news/lots-of-people-are-discovering-the-joy-of-birding-from-home-during-lockdown/ (2020).

[25] Zhang, G. Spatial and Temporal Patterns in Volunteer Data Contribution Activities: A Case Study of eBird. *ISPRS Inter. J. of Geo-Information* **9**, 597 (2020).

[26] Haider, N. et al. Lockdown measures in response to COVID-19 in nine sub-Saharan African countries. *BMJ Glob. Health* **5**, e003319 (2020). 33028699 10.1136/bmjgh-2020-003319 PMC7542624

[27] Sánchez-Clavijo, L. M. et al. Differential reporting of biodiversity in two citizen science platforms during COVID-19 lockdown in Colombia. *Biol. Conserv.* **256**, 109077 (2021). 35702146 10.1016/j.biocon.2021.109077 PMC9186113

[28] Pagés, C. *et al. From Lockdown to Reopening: Strategic Considerations for the Resumption of Activities in Latin America and the Caribbean within the Framework of Covid-19*. (2020).

[29] Kirby, T. South America prepares for the impact of COVID-19. *Lancet Respir. Med.*10.1016/S2213-2600(20)30218-6 (2020). 32359412 10.1016/S2213-2600(20)30218-6 PMC7190305

[30] Lau, H. *et al.* The positive impact of lockdown in Wuhan on containing the COVID-19 outbreak in China. *Journal of Travel Medicine* **27**, taaa037 (2020). 10.1093/jtm/taaa037 PMC7184469 32181488

[31] Balagtas See, A. Inside One of the World’s Longest COVID-19 Lockdowns. *TIME* https://time.com/5945616/covid-philippines-pandemic-lockdown/ (2021).

[32] Chan, T.-C. et al. Effectiveness of controlling COVID-19 epidemic by implementing soft lockdown policy and extensive community screening in Taiwan. *Sci. Rep.* **12**, 12053 (2022). 35835796 10.1038/s41598-022-16011-x PMC9282154

[33] Chhim, S. et al. Descriptive assessment of COVID-19 responses and lessons learnt in Cambodia, January 2020 to June 2022. *BMJ Glob. Health* **8**, e011885 (2023). 37137538 10.1136/bmjgh-2023-011885 PMC10163327

[34] GBIF.org. Search datasets. https://www.gbif.org/dataset/search?type=OCCURRENCE (2025).

[35] Rosenblatt, C. J. *et al.* Highly specialized recreationists contribute the most to the citizen science project eBird. *Ornithological Applications* **124**, duac008 (2022).

[36] UN DESA. *Inequality in a Rapidly Changing World.* (UN. Department of Economic and Social Affairs, 2020).

[37] OurWorldInData.org. Mobile phone subscriptions per 100 people. *Our World in Data* https://ourworldindata.org/grapher/mobile-cellular-subscriptions-per-100-people (2023).

[38] Johnson, N. et al. The use of digital platforms for community-based monitoring. *BioScience* **71**, 452–466 (2021). 33986630 10.1093/biosci/biaa162 PMC8106997

[39] Reyes-García, V. et al. Data sovereignty in community-based environmental monitoring: toward equitable environmental data governance. *BioScience* **72**, 714–717 (2022). 35923191 10.1093/biosci/biac048 PMC9343228

[40] Perino, A. et al. Biodiversity post-2020: Closing the gap between global targets and national-level implementation. *Conservation Letters* **15**, e12848 (2022).

[41] Sterner, B. & Elliott, S. How data governance principles influence participation in biodiversity science. *Sci. Culture* **33**, 366–391 (2024).

[42] Forrester, N. How to manage when your fieldwork is cancelled. *Nature*10.1038/d41586-020-03368-0 (2020). 33247292 10.1038/d41586-020-03368-0

[43] de Blocq, A. Providing relief and resilience for Community Bird Guides in South Africa faced with national lockdowns and tourism declines. https://panorama.solutions/en/solution/providing-relief-and-resilience-community-bird-guides-south-africa-faced-national-lockdowns (2022).

[44] Bowler, D. E. et al. Decision-making of citizen scientists when recording species observations. *Sci. Rep.* **12**, 11069 (2022). 35773384 10.1038/s41598-022-15218-2 PMC9245884

[45] Chadwick, F. J., Haydon, D. T., Husmeier, D., Ovaskainen, O. & Matthiopoulos, J. LIES of omission: complex observation processes in ecology. *Trends Ecol. Evolut.* **39**, 368–380 (2024). 10.1016/j.tree.2023.10.009 37949794

[46] GBIF.org. GBIF Occurrence Download. 10.15468/dl.uzt932 (2025).

[47] GBIF.org. Occurrence download formats. https://techdocs.gbif.org/en/data-use/download-formats (2025).

[48] Buchta, C. & Hornik, K. ISOcodes: Selected ISO Codes. R package version 2024.02.12 (2022).

[49] GBIF.org. Global data trends. https://www.gbif.org/analytics/global (2025).

[50] Helwig, NE. _npreg: Nonparametric Regression via Smoothing Splines_. R package version 1.1.0, https://CRAN.R-project.org/package=npreg (2024)

[51] R Core Team. R: The R Project for Statistical Computing. https://www.R-project.org/ (2024)

[52] Google LLC. COVID-19 Community Mobility Report. https://support.google.com/covid19-mobility (2025).

[53] Our World In Data. Data on COVID-19 (coronavirus) by Our World in Data. https://docs.owid.io/projects/covid/en/latest/dataset.html (2025).

[54] Massicotte, P., South, A. & Hufkens, K. rnaturalearth: World Map Data from Natural Earth. R package version 1.0.1 https://CRAN.R-project.org/package=rnaturalearth (2023).

[55] Hartig, F. DHARMa: Residual Diagnostics for Hierarchical (Multi-Level / Mixed) Regression Models. R package version 0.4.6 https://CRAN.R-project.org/package=DHARMa (2022).

[56] Pinheiro, J, Bates, D & R Core Team. nlme: Linear and Nonlinear Mixed Effects Models. R package version 3.1–165 https://CRAN.R-project.org/package=nlme (2024).

[57] Fox, J. & Weisberg, S. Visualizing Fit and Lack of Fit in Complex Regression Models with Predictor Effect Plots and Partial Residuals. *J. Stat. Software* **87**, 1–27 (2018).

[58] Fox, J. & Weisberg, S. *An R Companion to Applied Regression* (SAGE Publications, 2019).

