# Supplementary material for: Global impact of the COVID-19 lockdown on biodiversity data collection
Source: Sci Rep. 2025 Mar 13;15:8767. doi: 10.1038/s41598-025-93275-z (PMC11906878; doi:10.1038/s41598-025-93275-z)
Supplement: Supplementary file 1 — Supplementary Information. [file 41598_2025_93275_MOESM1_ESM.pdf]

Global impact of the COVID-19 lockdown on biodiversity data collection

Stephanie Roilo[1,2]*, Jan O. Engler[3], Anna F. Cord[1,2]

[1] Chair of Computational Landscape Ecology, TUD Dresden University of Technology, Helmholtzstr. 10, 10169 Dresden, Germany.

[2] Agro-Ecological Modeling Group, Institute of Crop Science and Resource Conservation, University of Bonn, Niebuhrstr. 1a, 53113 Bonn, Germany.

[3] AviCon - Forschung & Planung, 90765 Fürth, Germany.

* Corresponding author: Stephanie Roilo, stephanie.roilo@uni-bonn.de

SUPPLEMENTARY INFORMATION

Table S1: List of the 129 countries and dependent territories included in the linear regression, with the number of GBIF records (tagged as ‘human observation’) collected between March 15th and May 1st 2019, and in the same period in 2020 (first global lockdown period), the economic class of the country or dependent territory, the mean stringency index and mean change in park visitors during the lockdown. Change_records quantifies the percent change in GBIF records collected during the lockdown compared to the same period in 2019.

Country name	Country code	No. of GBIF records 15/03/2019 – 01/05/2029	No. of GBIF records 15/03/2020 – 01/05/2020	Economic class	Stringency index	Change in park visitors	Change_records
Aruba	AW	3,255	3,363	3. Developing region	70.76	-48.27	3.32
Afghanistan	AF	14	12	4. Least developed region	70.25	-10.21	-14.29
Angola	AO	85	109	4. Least developed region	69.67	-30.06	28.24
United Arab Emirates	AE	25,400	17,286	3. Developing region	78.12	-61.48	-31.94
Argentina	AR	122,918	77,363	2. Emerging region	92.38	-81.35	-37.06
Australia	AU	674,013	677,014	1. Developed region	65.18	-31.25	0.45
Austria	AT	48,054	83,369	1. Developed region	78.72	-24.69	73.49
Belgium	BE	144,935	132,730	1. Developed region	79.22	-18.44	-8.42
Benin	BJ	6,425	3,722	4. Least developed region	56.91	0.06	-42.07
Burkina Faso	BF	9	15	4. Least developed region	82.87	-14.77	66.67
Bangladesh	BD	5,865	13,352	4. Least developed region	83.68	-27.27	127.66
Bulgaria	BG	14,248	11,920	1. Developed region	71.16	-35.10	-16.34
Bahrain	BH	806	7	3. Developing region	71.91	-39.35	-99.13
Bahamas	BS	14,213	6,115	3. Developing region	88.06	-43.40	-56.98

Country name	Country code	No. of GBIF records 15/03/2019 – 01/05/2029	No. of GBIF records 15/03/2020 – 01/05/2020	Economic class	Stringency index	Change in park visitors	Change_records
Bosnia and Herzegovina	BA	433	418	3. Developing region	86.08	-10.46	-3.46
Belarus	BY	3,316	10,923	3. Developing region	21.47	12.92	229.40
Belize	BZ	179,240	93,276	3. Developing region	65.93	-43.19	-47.96
Bolivia	BO	10,446	4,349	2. Emerging region	91.67	-72.67	-58.37
Brazil	BR	131,601	158,812	2. Emerging region	72.20	-51.02	20.68
Barbados	BB	2,767	447	3. Developing region	71.93	-55.19	-83.85
Botswana	BW	6,250	4,016	3. Developing region	74.36	-17.19	-35.74
Canada	CA	1,897,729	2,437,508	1. Developed region	71.74	-6.13	28.44
Switzerland	CH	116,468	184,089	1. Developed region	71.34	13.60	58.06
Chile	CL	52,261	38,486	2. Emerging region	70.74	-60.75	-26.36
Côte d'Ivoire	CI	522	808	3. Developing region	73.77	-17.13	54.79
Cameroon	CM	933	242	3. Developing region	61.13	-18.33	-74.06
Colombia	CO	419,969	186,983	3. Developing region	79.36	-65.46	-55.48
Republic of Cabo Verde	CV	1,046	392	3. Developing region	68.69	-48.04	-62.52
Costa Rica	CR	341,068	187,024	2. Emerging region	72.61	-57.08	-45.17
Czech Republic	CZ	48,380	97,599	1. Developed region	71.43	-0.10	101.73
Germany	DE	335,329	513,083	1. Developed region	72.94	28.40	53.01
Denmark	DK	351,658	483,242	1. Developed region	70.50	84.75	37.42

Country name	Country code	No. of GBIF records 15/03/2019 – 01/05/2029	No. of GBIF records 15/03/2020 – 01/05/2020	Economic class	Stringency index	Change in park visitors	Change_records
Dominican Republic	DO	5,769	4,372	3. Developing region	88.10	-58.67	-24.22
Ecuador	EC	109,454	30,973	3. Developing region	91.94	-73.79	-71.70
Egypt	EG	7,995	1,197	2. Emerging region	74.34	-40.33	-85.03
Spain	ES	707,051	516,025	1. Developed region	80.84	-78.79	-27.02
Estonia	EE	63,656	67,519	1. Developed region	70.31	21.10	6.07
Finland	FI	88,207	132,950	1. Developed region	68.60	52.25	50.72
Fiji	FJ	3,032	585	3. Developing region	79.40	-18.48	-80.71
France	FR	1,497,171	1737,844	1. Developed region	86.51	-62.02	16.08
Gabon	GA	219	10	3. Developing region	71.87	-17.94	-95.43
United Kingdom	GB	823,020	799,004	1. Developed region	71.77	-15.48	-2.92
Georgia	GE	10,512	2,769	3. Developing region	91.32	-25.48	-73.66
Ghana	GH	6,229	1,085	3. Developing region	67.30	-23.88	-82.58
Greece	GR	55,352	26,291	1. Developed region	80.83	-36.08	-52.50
Guatemala	GT	98,830	58,485	3. Developing region	91.34	-47.06	-40.82
Hong Kong	HK	29,516	35,350	3. Developing region	61.54	-16.40	19.77
Honduras	HN	55,224	38,921	3. Developing region	98.01	-53.23	-29.52
Croatia	HR	7,882	11,783	1. Developed region	89.20	-14.98	49.49
Haiti	HT	3,415	1,066	4. Least developed region	82.31	-17.73	-68.78
Hungary	HU	22,114	23,136	1. Developed region	74.05	-9.46	4.62

Country name	Country code	No. of GBIF records 15/03/2019 – 01/05/2029	No. of GBIF records 15/03/2020 – 01/05/2020	Economic class	Stringency index	Change in park visitors	Change_records
Indonesia	ID	10,217	4,637	2. Emerging region	60.99	-33.38	-54.61
India	IN	623,379	601,807	2. Emerging region	90.41	-48.35	-3.46
Ireland	IE	33,795	36,680	1. Developed region	78.94	-11.54	8.54
Iraq	IQ	1,412	170	3. Developing region	92.09	-38.38	-87.96
Israel	IL	138,929	90,787	1. Developed region	85.57	-49.04	-34.65
Italy	IT	79,181	59,716	1. Developed region	88.49	-78.38	-24.58
Jamaica	JM	8,995	3,248	3. Developing region	82.33	-48.96	-63.89
Jordan	JO	2,432	538	3. Developing region	94.31	-55.08	-77.88
Japan	JP	201,491	46,895	1. Developed region	44.00	3.10	-76.73
Kazakhstan	KZ	1,836	1,291	3. Developing region	85.28	-20.83	-29.68
Kenya	KE	30,680	16,427	2. Emerging region	82.33	-24.71	-46.46
Kyrgyzstan	KG	49	112	3. Developing region	85.44	-27.17	128.57
Cambodia	KH	4,855	7,226	4. Least developed region	62.42	-25.25	48.84
Republic of Korea	KR	16,144	18,632	2. Emerging region	66.77	35.65	15.41
Kuwait	KW	5,119	2,610	3. Developing region	85.86	-52.88	-49.01
Lao PDR	LA	1,828	4,374	4. Least developed region	73.50	-14.25	139.28
Lebanon	LB	1,955	1,351	3. Developing region	82.47	-39.94	-30.90
Libya	LY	9	3	3. Developing region	85.36	-23.46	-66.67
Sri Lanka	LK	24,328	23,772	3. Developing region	89.85	-43.17	-2.29

**Country name**	**Country code**	**No. of GBIF records 15/03/2019 – 01/05/2029**	**No. of GBIF records 15/03/2020 – 01/05/2020**	**Economic class**	**Stringency index**	**Change in park visitors**	**Change_records**
Lithuania	LT	8,168	11,471	1. Developed region	80.69	33.42	40.44
Luxembourg	LU	15,723	20,466	1. Developed region	76.62	-28.73	30.17
Latvia	LV	2,653	1,808	1. Developed region	60.18	20.35	-31.85
Morocco	MA	36,752	1,873	3. Developing region	89.04	-63.56	-94.90
Moldova	MD	2,820	2,743	3. Developing region	80.38	-33.90	-2.73
Mexico	MX	345,709	225,454	2. Emerging region	65.28	-41.04	-34.79
Mali	ML	6	31	4. Least developed region	65.62	-6.38	416.67
Malta	MT	2,703	1,456	1. Developed region	80.27	-35.85	-46.13
Myanmar	MM	3,975	3,061	4. Least developed region	68.44	-14.88	-22.99
Mongolia	MN	3,679	8,610	3. Developing region	63.25	-2.48	134.03
Mozambique	MZ	4,766	950	4. Least developed region	47.47	-23.48	-80.07
Mauritius	MU	442	69	3. Developing region	71.68	-73.44	-84.39
Malaysia	MY	40,964	15,227	3. Developing region	74.71	-55.54	-62.83
Namibia	NA	18,807	6,636	3. Developing region	62.97	-32.04	-64.72
Niger	NE	86	3	4. Least developed region	57.23	-17.65	-96.51
Nigeria	NG	11,081	9,248	2. Emerging region	69.45	-34.60	-16.54
Nicaragua	NI	10,740	23,653	3. Developing region	18.07	-19.65	120.23
Netherlands	NL	1,037,172	1,355,513	1. Developed region	75.61	27.50	30.69
Norway	NO	393,759	448,288	1. Developed region	73.69	47.25	13.85

Country name	Country code	No. of GBIF records 15/03/2019 – 01/05/2029	No. of GBIF records 15/03/2020 – 01/05/2020	Economic class	Stringency index	Change in park visitors	Change_records
Nepal	NP	9,714	14,016	4. Least developed region	87.17	-43.17	44.29
New Zealand	NZ	70,381	106,813	1. Developed region	85.25	-61.98	51.76
Oman	OM	2,012	444	3. Developing region	83.97	-45.21	-77.93
Pakistan	PK	2,205	7,207	2. Emerging region	87.04	-29.13	226.85
Panama	PA	130,082	45,173	3. Developing region	82.48	-67.90	-65.27
Peru	PE	58,303	19,918	2. Emerging region	93.90	-72.75	-65.84
Philippines	PH	15,080	10,555	2. Emerging region	98.13	-50.19	-30.01
Papua New Guinea	PG	243	174	3. Developing region	66.18	-11.90	-28.40
Poland	PL	36,261	40,103	1. Developed region	75.19	-27.00	10.60
Portugal	PT	201,502	111,358	1. Developed region	80.94	-62.10	-44.74
Paraguay	PY	5,538	5,124	2. Emerging region	82.93	-59.00	-7.48
Qatar	QA	1,145	244	3. Developing region	80.61	-41.44	-78.69
Romania	RO	16,125	9,238	1. Developed region	81.46	-43.33	-42.71
Russian Federation	RU	61,039	96,387	2. Emerging region	77.02	-14.81	57.91
Rwanda	RW	7,301	1,941	4. Least developed region	84.35	-12.33	-73.41
Saudi Arabia	SA	2,452	619	1. Developed region	87.52	-58.10	-74.76
Senegal	SN	2,770	1,813	4. Least developed region	71.82	-25.48	-34.55
Singapore	SG	16,718	12,243	3. Developing region	63.73	-36.96	-26.77
El Salvador	SV	11,072	8,140	3. Developing region	86.25	-61.56	-26.48

Country name	Country code	No. of GBIF records 15/03/2019 – 01/05/2029	No. of GBIF records 15/03/2020 – 01/05/2020	Economic class	Stringency index	Change in park visitors	Change_records
Serbia	RS	11,570	10,995	3. Developing region	95.37	-42.94	-4.97
Slovakia	SK	7,178	21,124	1. Developed region	76.27	4.15	194.29
Slovenia	SI	6,062	10,706	1. Developed region	81.94	-22.25	76.61
Sweden	SE	1,016,904	1,125,512	1. Developed region	58.79	50.21	10.68
Togo	TG	122	129	4. Least developed region	66.32	-13.02	5.74
Thailand	TH	52,148	55,588	2. Emerging region	68.46	-42.38	6.60
Tajikistan	TJ	51	40	3. Developing region	27.12	-9.27	-21.57
Trinidad and Tobago	TT	9,043	6,097	3. Developing region	74.33	-49.08	-32.58
Turkey	TR	40,853	39,470	2. Emerging region	71.41	-44.02	-3.39
Taiwan	TW	261,659	314,162	1. Developed region	31.25	-3.90	20.07
Tanzania	TZ	10,445	6,622	4. Least developed region	45.47	-11.08	-36.60
Uganda	UG	3,508	3,159	4. Least developed region	78.76	-19.52	-9.95
Ukraine	UA	26,989	41,085	3. Developing region	87.35	-7.40	52.23
Uruguay	UY	21,731	10,094	2. Emerging region	62.85	-68.83	-53.55
United States	US	14,691,099	17,107,121	1. Developed region	70.64	-10.48	16.45
Venezuela	VE	23,012	23,225	2. Emerging region	81.50	-51.77	0.93
Vietnam	VN	11,403	2,661	2. Emerging region	77.88	-30.21	-76.66
Yemen	YE	88	4	4. Least developed region	55.79	-9.35	-95.45
South Africa	ZA	268,952	191,432	2. Emerging region	79.01	-38.83	-28.82

Country name	Country code	No. of GBIF records 15/03/2019 – 01/05/2029	No. of GBIF records 15/03/2020 – 01/05/2020	Economic class	Stringency index	Change in park visitors	Change_records
Zambia	ZM	3,409	5,337	4. Least developed region	48.06	-4.56	56.56

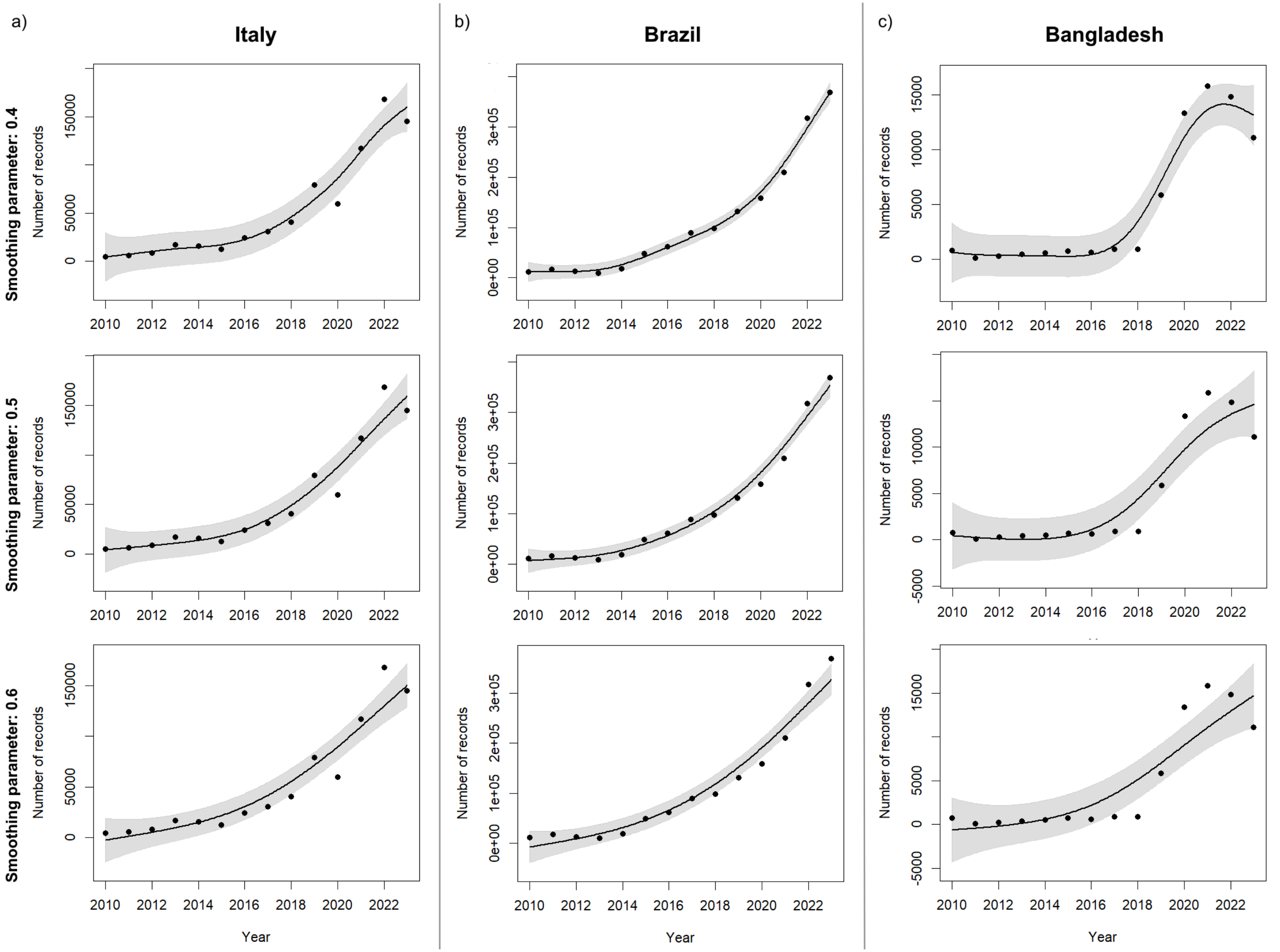

Figure S1: Smoothing splines approximating GBIF data trends in Italy (a), Brazil (b) and Bangladesh (c). The smoothing splines were fitted to the number of georeferenced GBIF records tagged as 'human observation' collected between March 15th and May 1st of the years 2010 to 2023; these data counts are shown as black dots in the plots. The grey shading indicates the estimated confidence interval (at the 0.95 level) of the smoothing spline. For each country, three smoothing splines were fitted with varying smoothing parameter of 0.4 (top row), 0.5 (middle row) and 0.6 (bottom row); lower values of the smoothing parameter generate more flexible splines.

Table S2: List of the 129 countries and dependent territories included in the linear regression, with the number of GBIF records (georeferenced and tagged as ‘human observation’) collected during the lockdown in 2020 (between March 15th and May 1st), and the predicted number of records as modelled by three smoothing splines approximating GBIF data trends in each country. The smoothing splines were fitted to the number of records collected between March 15th and May 1st every year from 2010 to 2023. The three smoothing splines were fitted with varying smoothing parameter (spar) of 0.4, 0.5 and 0.6; lower values of spar generate more flexible splines. Red shading indicates that the smoothing spline predicted a significantly (at the 0.95 level) higher number of records than observed; blue shading indicates that the smoothing spline predicted a significantly (at the 0.95 level) lower number of records than observed.

Country name	Country code	No. of GBIF records, 2020 lockdown	Predicted no. of records, spar=0.4	Lower CI, spar=0.4	Upper CI, spar=0.4	Predicted no. of records, spar=0.5	Lower CI, spar=0.5	Upper CI, spar=0.5	Predicted no. of records, spar=0.6	Lower CI, spar=0.6	Upper CI, spar=0.6
Aruba	AW	3,363	3,583	2,401	4,765	4,041	2,915	5,167	4,315	3,321	5,309
Afghanistan	AF	12	65	-395	525	77	-313	467	118	-212	448
Angola	AO	109	15	-447	477	251	-229	731	464	-2	930
United Arab Emirates	AE	17,286	25,448	20,550	30,346	24,885	20,639	29,131	24,215	20,601	27,829
Argentina	AR	77,363	121,828	100,886	142,770	129,722	111,788	147,656	135,050	118,248	151,852
Australia	AU	677,014	767,728	705,758	829,698	785,123	730,887	839,359	801,946	750,096	853,796
Austria	AT	83,369	71,123	61,947	80,299	75,242	65,892	84,592	81,133	68,605	93,661
Belgium	BE	132,730	194,521	92,867	296,175	244,202	144,688	343,716	264,538	171,104	357,972
Benin	BJ	3,722	3,488	164	6,812	4,464	1,380	7,548	5,049	2,173	7,925
Burkina Faso	BF	15	219	7	431	283	103	463	325	171	479
Bangladesh	BD	13,352	11,117	9,213	13,021	9,759	7,467	12,051	9,045	6,795	11,295
Bulgaria	BG	11,920	18,974	14,786	23,162	20,338	16,696	23,980	21,541	17,985	25,097

Country name	Country code	No. of GBIF records, 2020 lockdown	Predicted no. of records, spar=0.4	Lower CI, spar=0.4	Upper CI, spar=0.4	Predicted no. of records, spar=0.5	Lower CI, spar=0.5	Upper CI, spar=0.5	Predicted no. of records, spar=0.6	Lower CI, spar=0.6	Upper CI, spar=0.6
Bahrain	BH	7	513	223	803	526	302	750	535	351	719
Bahamas	BS	6,115	10,125	7,305	12,945	11,162	8,298	14,026	11,318	8,680	13,956
Bosnia and Herzegovina	BA	418	509	227	791	669	365	973	763	483	1,043
Belarus	BY	10,923	8,800	6,994	10,606	9,233	7,685	10,781	9,865	8,221	11,509
Belize	BZ	93,276	126,356	98,006	154,706	126,275	99,207	153,343	120,800	95,860	145,740
Bolivia, Plurinational State of	BO	4,349	7,798	4,520	11,076	8,539	5,467	11,611	8,707	5,813	11,601
Brazil	BR	158,812	170,452	157,100	183,804	181,189	166,427	195,951	190,728	171,442	210,014
Barbados	BB	447	1,471	697	2,245	1,717	959	2,475	1,812	1,150	2,474
Botswana	BW	4,016	5,162	3,324	7,000	6,199	4,365	8,033	6,805	5,123	8,487
Canada	CA	2,437,508	2,511,701	2,322,843	2,700,559	2,459,028	2,270,806	2,647,250	2,430,618	2,256,248	2,604,988
Switzerland	CH	184,089	160,719	145,379	176,059	153,402	138,988	167,816	148,401	134,695	162,107
Chile	CL	38,486	53,295	43,559	63,031	59,542	50,000	69,084	64,537	54,527	74,547
Côte d'Ivoire	CI	808	844	-704	2,392	1,263	-153	2,679	1,370	136	2,604
Cameroon	CM	242	455	-5,159	6,069	699	-3,861	5,259	1,157	-2,645	4,959
Colombia	CO	186,983	348,066	251,356	444,776	365,341	283,491	447,191	370,247	301,669	438,825
Cabo Verde	CV	392	793	-669	2,255	1,277	-7	2,561	1,549	433	2,665
Costa Rica	CR	187,024	294,753	232,613	356,893	323,776	267,398	380,154	343,280	290,946	395,614

Country name	Country code	No. of GBIF records, 2020 lockdown	Predicted no. of records, spar=0.4	Lower CI, spar=0.4	Upper CI, spar=0.4	Predicted no. of records, spar=0.5	Lower CI, spar=0.5	Upper CI, spar=0.5	Predicted no. of records, spar=0.6	Lower CI, spar=0.6	Upper CI, spar=0.6
Czechia	CZ	97,599	91,461	85,267	97,655	91,100	83,264	98,936	93,780	81,856	105,704
Germany	DE	513,083	459,962	426,632	493,292	448,395	415,663	481,127	446,275	413,241	479,309
Denmark	DK	483,242	429,864	398,240	461,488	418,985	391,137	446,833	414,519	389,799	439,239
Dominican Republic	DO	4,372	6,382	2,756	10,008	8,576	5,040	12,112	10,166	6,794	13,538
Ecuador	EC	30,973	74,545	48,873	100,217	86,557	62,327	110,787	94,262	72,378	116,146
Egypt	EG	1,197	4,377	1,099	7,655	6,413	2,643	10,183	7,333	3,829	10,837
Spain	ES	516,025	748,687	635,969	861,405	772,686	680,840	864,532	790,513	701,009	880,017
Estonia	EE	67,519	62,909	56,451	69,367	62,943	56,815	69,071	61,666	55,258	68,074
Finland	FI	132,950	102,515	87,229	117,801	94,726	80,708	108,744	89,795	76,959	102,631
Fiji	FJ	585	1,214	462	1,966	1,261	611	1,911	1,273	735	1,811
France	FR	1,737,844	1631,718	1490,716	1,772,720	1,452,289	1,243,137	1,661,441	1,298,870	1,057,944	1,539,796
Gabon	GA	10	169	21	317	204	84	324	233	129	337
United Kingdom	GB	799,004	911,951	802,719	1,021,183	920,303	831,059	1,009,547	940,151	851,743	1,028,559
Georgia	GE	2,769	6,184	2,482	9,886	7,657	4,335	10,979	8,759	5,777	11,741
Ghana	GH	1,085	7,975	3,739	12,211	10,588	6,598	14,578	12,808	8,688	16,928
Greece	GR	26,291	46,034	34,852	57,216	49,568	40,072	59,064	52,362	43,674	61,050
Guatemala	GT	58,485	74,774	60,948	88,600	77,193	63,815	90,571	76,921	64,893	88,949

Country name	Country code	No. of GBIF records, 2020 lockdown	Predicted no. of records, spar=0.4	Lower CI, spar=0.4	Upper CI, spar=0.4	Predicted no. of records, spar=0.5	Lower CI, spar=0.5	Upper CI, spar=0.5	Predicted no. of records, spar=0.6	Lower CI, spar=0.6	Upper CI, spar=0.6
Hong Kong	HK	35,350	38,151	35,081	41,221	36,482	33,224	39,740	35,449	31,821	39,077
Honduras	HN	38,921	53,105	44,163	62,047	53,892	46,224	61,560	53,410	46,824	59,996
Croatia	HR	11,783	12,265	10,503	14,027	14,504	11,888	17,120	16,615	13,195	20,035
Haiti	HT	1,066	2,569	1,419	3,719	2,212	1,196	3,228	1,929	1,039	2,819
Hungary	HU	23,136	26,244	22,992	29,496	26,015	23,255	28,775	26,374	23,470	29,278
Indonesia	ID	4,637	6,932	4,074	9,790	8,678	5,502	11,854	9,828	6,872	12,784
India	IN	601,807	697,693	650,085	745,301	723,278	675,642	770,914	739,504	685,678	793,330
Ireland	IE	36,680	37,045	33,209	40,881	36,672	33,338	40,006	36,823	33,373	40,273
Iraq	IQ	170	915	255	1,575	1,104	430	1,778	1,091	477	1,705
Israel	IL	90,787	114,780	94,150	135,410	118,705	101,309	136,101	121,911	106,469	137,353
Italy	IT	59,716	85,938	68,324	103,552	87,658	73,168	102,148	89,662	76,386	102,938
Jamaica	JM	3,248	3,795	-393	7,983	6,049	1,895	10,203	8,071	3,961	12,181
Jordan	JO	538	1,431	-549	3,411	2,819	535	5,103	3,992	1,648	6,336
Japan	JP	46,895	80,929	26,391	135,467	76,502	31,524	121,480	71,758	34,556	108,960
Kazakhstan	KZ	1,291	1,926	634	3,218	2,770	1,360	4,180	3,473	2,047	4,899
Kenya	KE	16,427	36,503	24,719	48,287	40,492	30,124	50,860	41,959	33,193	50,725
Kyrgyzstan	KG	112	413	103	723	660	318	1,002	881	497	1,265
Cambodia	KH	7,226	8,917	6,079	11,755	10,840	7,884	13,796	12,074	9,288	14,860

Country name	Country code	No. of GBIF records, 2020 lockdown	Predicted no. of records, spar=0.4	Lower CI, spar=0.4	Upper CI, spar=0.4	Predicted no. of records, spar=0.5	Lower CI, spar=0.5	Upper CI, spar=0.5	Predicted no. of records, spar=0.6	Lower CI, spar=0.6	Upper CI, spar=0.6
Korea, Republic of	KR	18,632	20,279	13,933	26,625	21,865	15,821	27,909	22,876	17,546	28,206
Kuwait	KW	2,610	4,020	908	7,132	4,781	1,955	7,607	5,423	2,825	8,021
Lao People's Democratic Republic	LA	4,374	3,840	3,170	4,510	3,419	2,661	4,177	3,207	2,467	3,947
Lebanon	LB	1,351	2,026	1,470	2,582	1,881	1,415	2,347	1,784	1,372	2,196
Libya	LY	3	17	-17	51	14	-20	48	8	-24	40
Sri Lanka	LK	23,772	32,258	26,846	37,670	32,261	27,501	37,021	32,768	28,004	37,532
Lithuania	LT	11,471	11,655	10,461	12,849	11,581	10,521	12,641	11,799	10,483	13,115
Luxembourg	LU	20,466	19,353	17,249	21,457	18,333	16,275	20,391	17,632	15,598	19,666
Latvia	LV	1,808	2,941	2,319	3,563	3,367	2,741	3,993	3,695	3,059	4,331
Morocco	MA	1,873	14,093	1,645	26,541	20,542	7,656	33,428	24,951	13,161	36,741
Moldova, Republic of	MD	2,743	2,882	1,948	3,816	2,697	1,935	3,459	2,549	1,909	3,189
Mexico	MX	225,454	307,776	260,488	355,064	325,281	283,861	366,701	336,203	298,773	373,633
Mali	ML	31	19	-45	83	43	-17	103	62	8	116
Malta	MT	1,456	2,310	1,742	2,878	2,281	1,793	2,769	2,193	1,763	2,623
Myanmar	MM	3,061	2,897	1,891	3,903	2,952	1,944	3,960	2,776	1,798	3,754
Mongolia	MN	8,610	5,547	4,113	6,981	4,700	3,366	6,034	4,117	2,859	5,375
Mozambique	MZ	950	2,908	1,466	4,350	3,560	2,306	4,814	4,092	2,924	5,260

Country name	Country code	No. of GBIF records, 2020 lockdown	Predicted no. of records, spar=0.4	Lower CI, spar=0.4	Upper CI, spar=0.4	Predicted no. of records, spar=0.5	Lower CI, spar=0.5	Upper CI, spar=0.5	Predicted no. of records, spar=0.6	Lower CI, spar=0.6	Upper CI, spar=0.6
Mauritius	MU	69	192	-174	558	247	-77	571	271	-15	557
Malaysia	MY	15,227	25,947	15,407	36,487	30,662	19,718	41,606	33,512	23,686	43,338
Namibia	NA	6,636	17,437	11,533	23,341	17,971	13,041	22,901	17,912	13,830	21,994
Niger	NE	3	60	-34	154	77	-1	155	81	15	147
Nigeria	NG	9,248	14,284	10,062	18,506	12,754	8,748	16,760	11,059	7,211	14,907
Nicaragua	NI	23,653	25,385	20,511	30,259	25,494	21,094	29,894	26,167	22,061	30,273
Netherlands	NL	1,355,513	1,297,555	1,236,167	1,358,943	1,275,408	1,212,832	1,337,984	1,269,336	1,206,488	1,332,184
Norway	NO	448,288	415,466	394,078	436,854	405,860	386,970	424,750	399,338	382,558	416,118
Nepal	NP	14,016	15,358	13,838	16,878	17,346	15,112	19,580	19,260	16,256	22,264
New Zealand	NZ	106,813	107,264	99,220	115,308	105,701	97,967	113,435	106,139	96,753	115,525
Oman	OM	444	1,166	300	2,032	1,697	813	2,581	2,118	1,260	2,976
Pakistan	PK	7,207	5,401	4,079	6,723	4,662	3,372	5,952	4,249	3,049	5,449
Panama	PA	45,173	95,513	66,881	124,145	108,468	82,672	134,264	116,043	93,295	138,791
Peru	PE	19,918	43,234	24,724	61,744	48,703	32,197	65,209	51,629	37,239	66,019
Philippines	PH	10,555	13,767	10,539	16,995	16,495	12,687	20,303	18,683	14,681	22,685
Papua New Guinea	PG	174	91	-1,283	1,465	343	-1,025	1,711	423	-841	1,687
Poland	PL	40,103	46,123	37,965	54,281	53,787	43,703	63,871	61,334	48,138	74,530
Portugal	PT	111,358	181,793	148,303	215,283	194,369	166,391	222,347	202,732	178,390	227,074

Country name	Country code	No. of GBIF records, 2020 lockdown	Predicted no. of records, spar=0.4	Lower CI, spar=0.4	Upper CI, spar=0.4	Predicted no. of records, spar=0.5	Lower CI, spar=0.5	Upper CI, spar=0.5	Predicted no. of records, spar=0.6	Lower CI, spar=0.6	Upper CI, spar=0.6
Paraguay	PY	5,124	8,212	5,632	10,792	8,658	6,392	10,924	8,957	7,051	10,863
Qatar	QA	244	820	342	1,298	1,185	647	1,723	1,449	915	1,983
Romania	RO	9,238	10,657	7,665	13,649	11,019	7,911	14,127	10,784	7,964	13,604
Russian Federation	RU	96,387	99,154	91,200	107,108	105,182	95,700	114,664	112,843	97,709	127,977
Rwanda	RW	1,941	4,087	2,259	5,915	3,824	2,376	5,272	3,566	2,350	4,782
Saudi Arabia	SA	619	253	-118,225	118,731	-963	-99,779	97,853	6,135	-77,947	90,217
Senegal	SN	1,813	1,939	873	3,005	2,687	1,395	3,979	3,172	1,936	4,408
Singapore	SG	12,243	20,408	16,180	24,636	21,272	17,792	24,752	21,713	18,591	24,835
El Salvador	SV	8,140	13,380	10,440	16,320	14,415	11,947	16,883	15,222	13,028	17,416
Serbia	RS	10,995	13,001	11,429	14,573	14,057	12,203	15,911	14,586	12,810	16,362
Slovakia	SK	21,124	18,879	15,307	22,451	17,320	13,306	21,334	16,747	12,831	20,663
Slovenia	SI	10,706	10,801	9,049	12,553	10,341	8,679	12,003	10,113	8,525	11,701
Sweden	SE	1,125,512	1,083,064	1,039,704	1,126,424	1,064,689	1,025,189	1,104,189	1,056,862	1,017,840	1,095,884
Togo	TG	129	25	-1,621	1,671	251	-1,109	1,611	420	-722	1,562
Thailand	TH	55,588	70,192	58,652	81,732	74,260	64,480	84,040	78,413	67,665	89,161
Tajikistan	TJ	40	725	343	1,107	917	563	1,271	1,114	718	1,510
Trinidad and Tobago	TT	6,097	7,542	3,814	11,270	8,892	5,658	12,126	9,648	6,878	12,418

Country name	Country code	No. of GBIF records, 2020 lockdown	Predicted no. of records, spar=0.4	Lower CI, spar=0.4	Upper CI, spar=0.4	Predicted no. of records, spar=0.5	Lower CI, spar=0.5	Upper CI, spar=0.5	Predicted no. of records, spar=0.6	Lower CI, spar=0.6	Upper CI, spar=0.6
Turkey	TR	39,470	49,846	43,716	55,976	53,748	47,736	59,760	57,414	50,344	64,484
Taiwan, Province of China	TW	314,162	315,443	304,049	326,837	315,228	304,940	325,516	317,764	302,986	332,542
Tanzania, United Republic of	TZ	6,622	12,782	268	25,296	18,219	6,235	30,203	21,543	10,921	32,165
Uganda	UG	3,159	6,728	2,258	11,198	7,465	3,321	11,609	7,756	3,760	11,752
Ukraine	UA	41,085	35,669	27,993	43,345	33,144	26,536	39,752	32,175	26,249	38,101
Uruguay	UY	10,094	17,928	13,014	22,842	18,446	14,574	22,318	18,849	15,477	22,221
United States	US	17,107,121	17,574,907	17,051,281	18,098,533	17,514,871	17,025,499	18,004,243	17,540,770	17,005,636	18,075,904
Venezuela, Bolivarian Republic of	VE	23,225	36,575	29,197	43,953	39,932	33,052	46,812	42,898	35,336	50,460
Viet Nam	VN	2,661	4,786	-24	9,596	6,703	1,839	11,567	8,047	3,697	12,397
Yemen	YE	4	109	-127	345	131	-59	321	146	-8	300
South Africa	ZA	191,432	262,726	221,352	304,100	262,265	226,673	297,857	256,164	225,210	287,118
Zambia	ZM	5,337	4,682	4,096	5,268	4,718	4,246	5,190	4,829	4,347	5,311
Zimbabwe	ZW	9,353	9,811	8,197	11,425	9,406	7,918	10,894	9,136	7,854	10,418

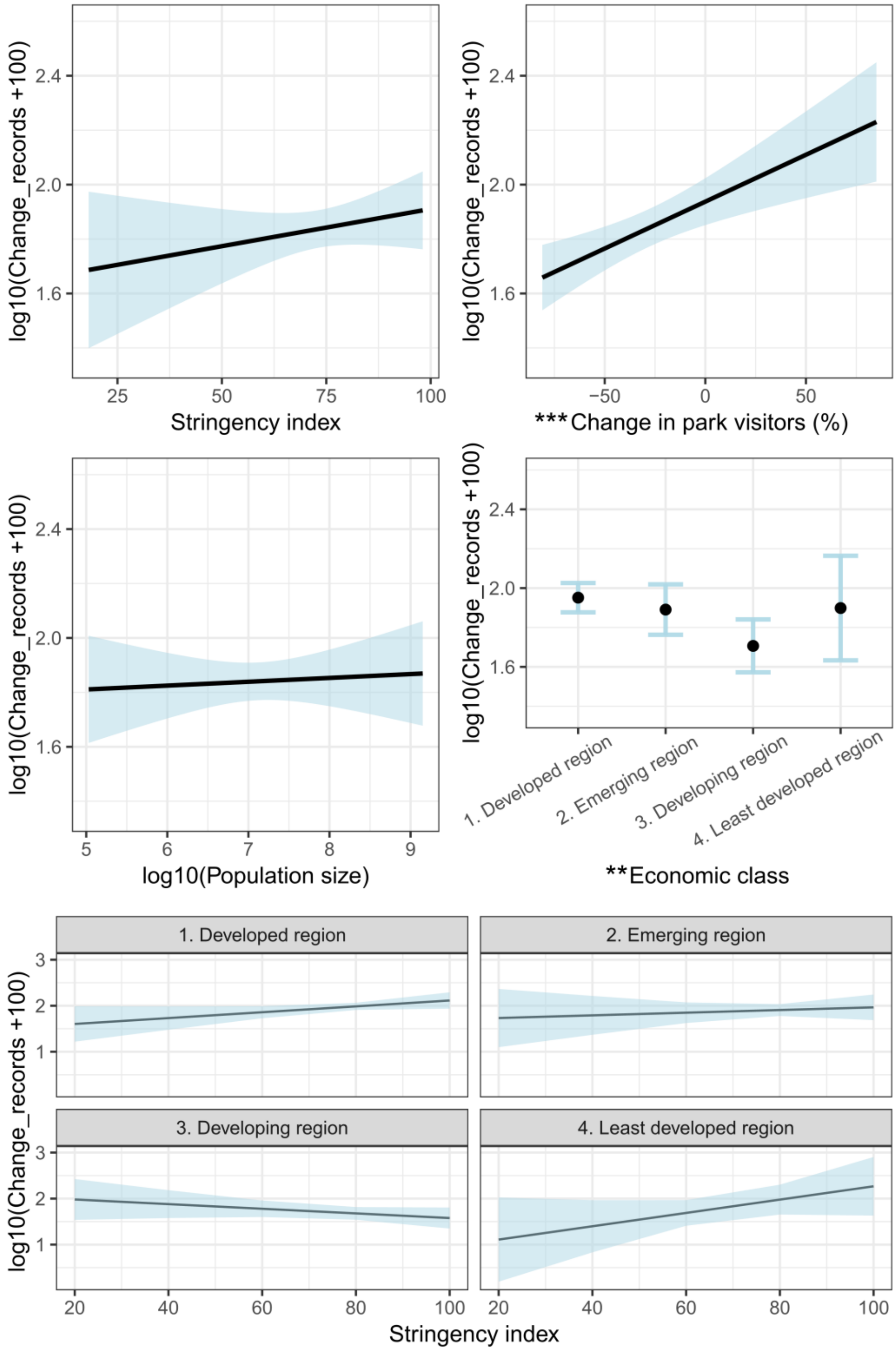

Figure S2: Conditional plots of the predictors of the generalised least squares regression model including the interaction term between *Stringency_index* and *Economic_class*. Relationships were graphed for each predictor with all other continuous covariates held at their means, or by weighting levels of the categorical covariate in proportion to sample size. The light-blue shading shows the confidence interval at the 0.95 level. Asterisks indicate significance of the predictor at the 0.01 (**) and 0.001 (***) levels, based on the estimated p-values from the model's analysis of variance.

Table S3: Number of mapped activity ranges of eBirders per country or dependent territory, calculated for the time periods March 15th to May 1st 2019 and March 15th to May 1st 2020. *D_stat* indicates the value of the two-sample Kolmogorov-Smirnov test statistic, and *p_value* the p-value of the test. For some countries, fewer than 30 eBirders were active between March 15th and May 1st 2019, so that the number of mapped activity ranges in 2019 is lower than 30. The number of mapped activity ranges can be lower in 2020 compared to 2019 if eBirders became inactive (i.e. did not record any observation) during the lockdown.

Country name	Country code	Number of activity ranges mapped in 2019	Number of activity ranges mapped in 2020	D_stat	p_value
United States	US	30	30	0.433	0.007
Canada	CA	30	29	0.425	0.006
Spain	ES	30	30	0.7	0
Australia	AU	30	27	0.537	0
United Kingdom	GB	30	29	0.52	0
Taiwan	TW	30	30	0.167	0.808
Portugal	PT	30	30	0.633	0
Israel	IL	30	29	0.594	0
Germany	DE	30	28	0.619	0
France	FR	30	30	0.667	0
India	IN	30	28	0.474	0.002
Costa Rica	CR	30	27	0.485	0.001
Mexico	MX	30	28	0.295	0.125
Brazil	BR	30	28	0.619	0
Argentina	AR	30	28	0.69	0
Peru	PE	30	20	0.7	0
Chile	CL	30	28	0.481	0.001
Thailand	TH	30	28	0.59	0
South Africa	ZA	30	24	0.6	0
Turkey	TR	30	30	0.633	0
Colombia	CO	30	27	0.489	0.001
Belize	BZ	30	26	0.362	0.038
Panama	PA	30	25	0.62	0
Guatemala	GT	30	27	0.641	0
Ecuador	EC	30	28	0.65	0
Honduras	HN	30	27	0.785	0

Country name	Country code	Number of activity ranges mapped in 2019	Number of activity ranges mapped in 2020	D_stat	p_value
Malaysia	MY	30	25	0.767	0
Morocco	MA	30	25	0.66	0
United Arab Emirates	AE	30	25	0.54	0
Hong Kong	HK	30	29	0.38	0.021
Nepal	NP	30	21	0.524	0.001
Rwanda	RW	30	12	0.517	0.013
Tanzania, United Republic of	TZ	30	19	0.447	0.012
Bangladesh	BD	30	20	0.25	0.293
Cambodia	KH	30	20	0.567	0.001
Myanmar	MM	30	15	0.3	0.265
Haiti	HT	17	16	0.412	0.068
Senegal	SN	8	4	0.5	0.479
Uganda	UG	25	15	0.24	0.393
Mozambique	MZ	16	13	0.534	0.021

Table S4: Number of eBird records collected between March 15th and May 1st 2019 in each country or dependent territory, and their proportion relative to the total number of records in GBIF tagged as 'human observation' for the same period. The economic class of each country is shown, and the countries selected for the eBird activity range analysis are highlighted in different colours per economic class (ten for each economic class).

Country name	Country code	No. of eBird records 15/03/2019 – 01/05/2019	Proportion of eBird records on GBIF records (%)	Economic class
United States	US	13,987,231	95%	1. Developed region
Canada	CA	1,823,949	96%	1. Developed region
India	IN	600,287	96%	2. Emerging region
Spain	ES	536,394	76%	1. Developed region
Australia	AU	478,421	71%	1. Developed region
United Kingdom	GB	387,040	47%	1. Developed region
Costa Rica	CR	328,840	96%	2. Emerging region
Colombia	CO	280,294	67%	3. Developing region
Mexico	MX	276,261	80%	2. Emerging region
Taiwan	TW	197,839	76%	1. Developed region
Belize	BZ	178,499	100%	3. Developing region
Portugal	PT	171,466	85%	1. Developed region
Israel	IL	129,271	93%	1. Developed region
Panama	PA	126,038	97%	3. Developing region
Brazil	BR	124,065	94%	2. Emerging region
Argentina	AR	114291	93%	2. Emerging region
Guatemala	GT	98,431	100%	3. Developing region
Ecuador	EC	97,146	89%	3. Developing region
Germany	DE	75,798	23%	1. Developed region
France	FR	65,694	4%	1. Developed region
Peru	PE	56,486	97%	2. Emerging region
Honduras	HN	54,278	98%	3. Developing region
Chile	CL	50,059	96%	2. Emerging region
Thailand	TH	48,259	93%	2. Emerging region
Sweden	SE	41,212	4%	1. Developed region
South Africa	ZA	40,854	15%	2. Emerging region
Turkey	TR	38,835	95%	2. Emerging region

Country name	Country code	No. of eBird records 15/03/2019 – 01/05/2019	Proportion of eBird records on GBIF records (%)	Economic class
New Zealand	NZ	38,066	54%	1. Developed region
Czech Republic	CZ	34,931	72%	1. Developed region
Malaysia	MY	34,112	83%	3. Developing region
Netherlands	NL	32,296	3%	1. Developed region
Morocco	MA	31,846	87%	3. Developing region
Finland	FI	30,919	35%	1. Developed region
Greece	GR	27,096	49%	1. Developed region
United Arab Emirates	AE	24,396	96%	3. Developing region
Japan	JP	24,026	12%	1. Developed region
Italy	IT	23,879	30%	1. Developed region
Venezuela	VE	22,908	100%	2. Emerging region
Uruguay	UY	21,070	97%	2. Emerging region
Poland	PL	20,348	56%	1. Developed region
Hong Kong	HK	20,122	68%	3. Developing region
Switzerland	CH	18,093	16%	1. Developed region
Ireland	IE	17,055	50%	1. Developed region
Belgium	BE	16,313	11%	1. Developed region
Sri Lanka	LK	15,679	64%	3. Developing region
Singapore	SG	14,755	88%	3. Developing region
Norway	NO	14,691	4%	1. Developed region
Kenya	KE	14,541	47%	2. Emerging region
Bahamas	BS	13,768	97%	3. Developing region
Philippines	PH	13,515	90%	2. Emerging region
Austria	AT	12,607	26%	1. Developed region
Denmark	DK	12,356	4%	1. Developed region
Russian Federation	RU	11,885	19%	2. Emerging region
Ukraine	UA	11,620	43%	3. Developing region
El Salvador	SV	10,958	99%	3. Developing region
Serbia	RS	10,384	90%	3. Developing region
Bulgaria	BG	10,291	72%	1. Developed region
Hungary	HU	10,112	46%	1. Developed region

Country name	Country code	No. of eBird records 15/03/2019 – 01/05/2019	Proportion of eBird records on GBIF records (%)	Economic class
Nicaragua	NI	10,101	94%	3. Developing region
Vietnam	VN	10,075	88%	2. Emerging region
Estonia	EE	9,819	15%	1. Developed region
Trinidad and Tobago	TT	8,930	99%	3. Developing region
Jamaica	JM	8,886	99%	3. Developing region
Nepal	NP	8,760	90%	4. Least developed region
Republic of Korea	KR	7,906	49%	2. Emerging region
Romania	RO	7,270	45%	1. Developed region
Rwanda	RW	7,239	99%	4. Least developed region
Tanzania	TZ	6,983	67%	4. Least developed region
Bolivia	BO	6,935	66%	2. Emerging region
Egypt	EG	6,795	85%	2. Emerging region
Georgia	GE	6,496	62%	3. Developing region
Bangladesh	BD	5,677	97%	4. Least developed region
Ghana	GH	5,658	91%	3. Developing region
Paraguay	PY	5,388	97%	2. Emerging region
Lithuania	LT	5,379	66%	1. Developed region
Indonesia	ID	5,282	52%	2. Emerging region
Dominican Republic	DO	4,953	86%	3. Developing region
Cambodia	KH	4,725	97%	4. Least developed region
Kuwait	KW	4,659	91%	3. Developing region
Slovakia	SK	4,289	60%	1. Developed region
Slovenia	SI	4,066	67%	1. Developed region
Myanmar	MM	3,932	99%	4. Least developed region
Croatia	HR	3,628	46%	1. Developed region
Namibia	NA	3,333	18%	3. Developing region
Aruba	AW	3,161	97%	3. Developing region
Botswana	BW	3,089	49%	3. Developing region
Haiti	HT	3,089	90%	4. Least developed region
Moldova	MD	2,804	99%	3. Developing region
Belarus	BY	2,763	83%	3. Developing region

Country name	Country code	No. of eBird records 15/03/2019 – 01/05/2019	Proportion of eBird records on GBIF records (%)	Economic class
Barbados	BB	2,739	99%	3. Developing region
Senegal	SN	2,596	94%	4. Least developed region
Uganda	UG	2,486	71%	4. Least developed region
Saudi Arabia	SA	2,390	97%	1. Developed region
Mozambique	MZ	2,375	50%	4. Least developed region
Malta	MT	2,305	85%	1. Developed region
Pakistan	PK	2,014	91%	2. Emerging region
Zimbabwe	ZW	1,892	26%	2. Emerging region
Jordan	JO	1,855	76%	3. Developing region
Latvia	LV	1,715	65%	1. Developed region
Lao PDR	LA	1,614	88%	4. Least developed region
Zambia	ZM	1,449	43%	4. Least developed region
Lebanon	LB	1,286	66%	3. Developing region
Iraq	IQ	1,270	90%	3. Developing region
Mongolia	MN	1,256	34%	3. Developing region
Qatar	QA	1,137	99%	3. Developing region
Kazakhstan	KZ	1,111	61%	3. Developing region
Republic of Cabo Verde	CV	932	89%	3. Developing region
Oman	OM	856	43%	3. Developing region
Bahrain	BH	805	100%	3. Developing region
Cameroon	CM	645	69%	3. Developing region
Luxembourg	LU	513	3%	1. Developed region
CÃ´te d'Ivoire	CI	436	84%	3. Developing region
Fiji	FJ	396	13%	3. Developing region
Nigeria	NG	338	3%	2. Emerging region
Bosnia and Herzegovina	BA	171	39%	3. Developing region
Papua New Guinea	PG	166	68%	3. Developing region
Mauritius	MU	128	29%	3. Developing region
Togo	TG	116	95%	4. Least developed region
Angola	AO	71	84%	4. Least developed region

**Country name**	**Country code**	**No. of eBird records 15/03/2019 – 01/05/2019**	**Proportion of eBird records on GBIF records (%)**	**Economic class**
Kyrgyzstan	KG	45	92%	3. Developing region
Niger	NE	43	50%	4. Least developed region
Yemen	YE	36	41%	4. Least developed region
Tajikistan	TJ	21	41%	3. Developing region
Gabon	GA	20	9%	3. Developing region
Mali	ML	6	100%	4. Least developed region
Afghanistan	AF	0	0%	4. Least developed region
Benin	BJ	0	0%	4. Least developed region
Burkina Faso	BF	0	0%	4. Least developed region
Libya	LY	0	0%	3. Developing region
